# A Fork Trap in the Chromosomal Termination Area Is Highly Conserved across All *Escherichia coli* Phylogenetic Groups

**DOI:** 10.3390/ijms22157928

**Published:** 2021-07-25

**Authors:** Daniel J. Goodall, Katie H. Jameson, Michelle Hawkins, Christian J. Rudolph

**Affiliations:** 1 Division of Biosciences, College of Health, Medicine and Life Sciences, Brunel University London, Uxbridge UB8 3PH, UK; Daniel.Goodall@brunel.ac.uk; 2 Department of Biology, University of York, Wentworth Way, York YO10 5DD, UK; katie.jameson@york.ac.uk (K.H.J.); michelle.hawkins@york.ac.uk (M.H.)

**Keywords:** replication fork trap, termination of DNA replication, bacterial chromosome, chromosomal architecture, *ter* terminator sequence, Tus protein, chromosomal over-replication

## Abstract

Termination of DNA replication, the final stage of genome duplication, is surprisingly complex, and failures to bring DNA synthesis to an accurate conclusion can impact genome stability and cell viability. In *Escherichia coli*, termination takes place in a specialised termination area opposite the origin. A ‘replication fork trap’ is formed by unidirectional fork barriers via the binding of Tus protein to genomic *ter* sites. Such a fork trap system is found in some bacterial species, but it appears not to be a general feature of bacterial chromosomes. The biochemical properties of fork trap systems have been extensively characterised, but little is known about their precise physiological roles. In this study, we compare locations and distributions of *ter* terminator sites in *E. coli* genomes across all phylogenetic groups, including *Shigella*. Our analysis shows that all *ter* sites are highly conserved in *E. coli*, with slightly more variability in the *Shigella* genomes. Our sequence analysis of *ter* sites and Tus proteins shows that the fork trap is likely to be active in all strains investigated. In addition, our analysis shows that the *dif* chromosome dimer resolution site is consistently located between the innermost *ter* sites, even if rearrangements have changed the location of the innermost termination area. Our data further support the idea that the replication fork trap has an important physiological role that provides an evolutionary advantage.

## 1. Introduction

In all domains of life, chromosome duplication initiates at origins of replication by the assembly of two multi-subunit ‘replisomes’. These replisome–DNA replication fork complexes (called ‘replication forks’ from here onwards for simplicity) move in opposite directions until they either merge with another replication fork or reach a chromosome end [1,2,3]. In bacteria, the number of fork fusions is restricted by the overall chromosomal architecture. The majority of bacteria carry a single circular chromosome that is replicated from a single origin called *oriC*. Bacterial chromosomes are therefore replicated by two replication forks, each duplicating one chromosomal half or ‘replichore’ [3,4,5].

**Figure 1:**
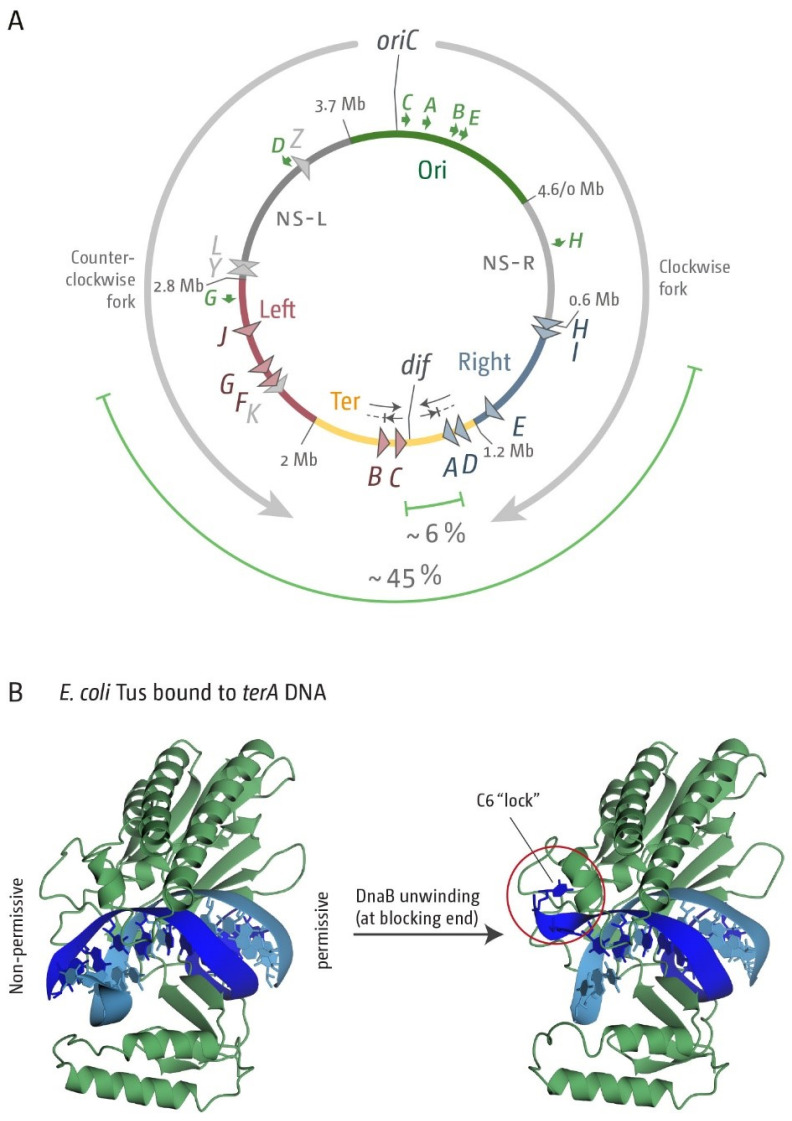
Chromosome structure and replication dynamics in *Escherichia coli*. (**A**) Schematic representation of the circular *E. coli* chromosome. Two replication forks are assembled at the origin (*oriC*) and move in opposite directions along the DNA (grey arrows) until they eventually approach one other and fuse within the terminus region diametrically opposite to *oriC*. A replication fork trap is formed in the terminus region via primary terminator sequences (*terA–J*). These are arranged as two opposed groups, with the red terminators oriented to block movement of the clockwise replication fork and the blue terminators oriented to block the anticlockwise fork. The total spanned fork trap area covered by *ter* sites, as well as the innermost termination area, are highlighted by green lines, with the fraction of the total genome size given in %. Secondary *ter* sites in the left-hand replichore with very low fork blocking activity [15] are shown in grey. Locations for *oriC* and the *dif* chromosome dimer resolution site are marked. The location of *rrn* operons, which are highly transcribed particularly under fast growth conditions, are shown by green arrows, with the arrow pointing in the direction of active transcription. Chromosomal macrodomains Ori, NS-R, NS-L, Right, Left and Ter are shown as described [7] and domain boundaries given in Mbp. (**B**) Crystal structure of the *ter*/Tus complex of *E. coli* indicating the blocking and permissive ends of the complex (left), and in the ‘locked’ conformation with DNA unwound at the blocking end and the C6 base of *ter* DNA bound to its specific binding pocket in Tus (right), which contributes significantly to the fork arrest activity of the complex.

In addition to the replichore structure, the *Escherichia coli* chromosome is further structured into four macrodomains, which are called Ori, Ter, Left and Right, as well as two more flexible and non-structured regions called NS-Left (NS-L) and NS-Right (NS-R), which flank the Ori macrodomain (Figure 1A) [6,7,8]. DNA replication is completed once the two replication forks merge opposite *oriC* in the terminus area of the chromosome, which is located in the Ter macrodomain. The main protein involved in the organisation of the Ter macrodomain is the DNA-binding protein MatP, which binds to *matS* sequences located in the Ter domain [9]. MatP is also involved in linking the Ter macrodomain to the Z-ring involved in septation [10].

Besides *matS*, additional genetic elements involved in the final stages of the cell cycle are located in the origin-distal half of the chromosome. For example, FtsK-Orienting Polar Sequences (KOPS) are found in a wide variety of bacterial species and direct FtsK DNA translocase towards the terminus region, where it plays an important role in chromosome segregation [11,12]. In addition, FtsK is required for the resolution of chromosomal dimers, which can form as a consequence of an odd number of recombination events. FtsK controls a site-specific recombination system that resolves chromosomal dimers via the *dif* chromosome dimer resolution site and the site-specific XerCD recombinase [11,12,13,14].

In *E. coli,* the chromosomal terminus area also contains a specialised ‘replication fork trap’ (Figure 1A). This system is generated by a series of polar blocks, which allow replisomes to pass in one direction, but block replisome progression in the other [16]. Ten non-palindromic *ter* sequences (*terA–J*) are symmetrically located either side of the terminus area of the chromosome (Figure 1A). If a 23 bp *ter* site is bound by Tus terminator protein, a polar block is generated (Figure 1B) due to the asymmetric binding of Tus [15,17]. In *E. coli* K-12, each replisome will replicate through the first five *ter* sites it encounters in the permissive direction by displacing Tus protein. However, the replisome will be paused by the first *ter*/Tus complex encountered in the non-permissive orientation on the other side of the termination area [3,5,15,16]. Forks are paused at *ter*/Tus complexes for a considerable period [18,19]. Indeed, in cells in which the replichore arrangement is skewed due to the presence of a second ectopic replication origin, which forces forks to be arrested at *ter*/Tus complexes every single cell cycle, very little indication is seen that forks proceed past the first *ter*/Tus complex encountered on a regular basis [3,20]. However, *ter*/Tus complexes can be overcome if forks are stalled for long periods of time [18,21], and it was suggested that the multiple *ter* sites introduce a level of redundancy, so that if a fork overcomes a *ter*/Tus complex, proceeding synthesis is arrested again at the next *ter*/Tus complex, in line with a reduced number of forks at the second *ter* sites *B* and *D* [15].

The fork trap system dictates that fork fusions take place between the innermost *ter* sites (*terA* and *C* in *E. coli*). However, in normally growing cells, the majority of fusions typically occur away from *ter*/Tus complexes, as demonstrated by labelling experiments [22], and, more recently, by marker frequency analysis produced by Whole Genome Sequencing (WGS) [23].

Recent analysis illustrated that replication fork traps are a specialised feature of a relatively small group of bacteria. They can be found in most Enterobacteriales, the Pseudoalteromonas and most Aeromonadales [24]. A similar fork trap system has been demonstrated to operate in the Gram-positive *Bacillus subtilis*. However, while working in a similar fashion, this fork trap system shows no significant sequence or structural similarity to the fork trap system in the Enterobacteriales, indicating that they have evolved via convergent evolution [16]. Convergent evolution often suggests a particularly important physiological function. However, phenotypes observed in cells in which only the replication fork trap is inactivated are very mild [25,26] unless other mutations are introduced.

In *E. coli,* the primary *ter* sites *A–J* span a significant area of the chromosome (almost 45%) (Figure 1A). In contrast, the fork trap system in *B. subtilis* is smaller (9.9%). More recently, four additional *ter* sites, *K*, *L*, *Y* and *Z*, were identified in *E. coli* [15]. However, fork pausing efficiencies for these four *ter* sites were established to be very low, and two of them, *terY* and *terZ*, are in fact in the blocking orientation for forks coming from *oriC* [15]. We will refer to these as secondary *ter* sites. In *E. coli,* the most significant levels of blocked forks were found at the four inner-most sites during normal chromosome replication, even though all primary *ter* sites can block fork progression if bound by Tus [15]. The innermost *ter* sites *A–D* therefore form the primary fork trap, which spans ~8% of the chromosome (Figure 1A), an area similar to the *B. subtilis* termination area. The area between the innermost *ter* sites *A* and *C* where forks will fuse spans approximately 6% of the chromosome.

In their recent study, Galli and co-workers based their conclusions about the presence of a replication fork trap on the presence of the terminator proteins Tus (*E. coli*) and RTP (*B. subtilis*) [24]. Here, we have directly investigated the replication fork trap area in a variety of different *E. coli* strains across all phylogroups, including Shigella, to analyse the variability of the 10 primary and four secondary *ter* sites. The majority of the past analysis has been conducted in *E. coli* K12 and, given the mild phenotype of cells lacking Tus, other phylogenetic groups might be expected to show considerable variation in the number and location of *ter* sites if the inactivation of the replication fork trap does not have a major impact on the cells.

Our analysis shows that all 10 primary *ter* sites are present in the *E. coli* genomes analysed, with only *Shigella* showing some variability. For the majority of *ter* sites in the various *E. coli* phylogenetic groups, the inner core matches the consensus sequence derived from the *ter* sequences in MG1655, strongly indicating that these *ter* sites are fully functional. We found that for all strains analysed, the *dif* chromosome dimer resolution site is consistently present next to *terC* within the innermost termination area. In Shigella, which has arisen multiple independent times from *E. coli* [27,28], we found some strains where the relative location of *dif* to the innermost *ter* sites differs. However, in these strains, specific *ter* sites are flipped, placing *dif* again in the innermost termination area. Finally, we extend our analysis to some *Salmonella* and *Klebsiella* genomes, showing that a fork trap is likely to be present. The fact that a distinct fork trap area is consistently maintained supports the idea that it provides a distinct evolutionary advantage and highlights the urgent need to establish what its specific physiological function is.

## 2. Material and Methods

### 2.1. Strains, Media and General Methods

For *Escherichia coli* K12 strains used in this study, see Table S1. Strains were constructed via P1*vir* transductions [29] or by single-step gene disruptions [30]. Luria broth (LB) and agar was modified from Luria and Burrous [31] as follows: 1% tryptone (Bacto™, BD Biosciences, Wokingham, Berkshire, UK), 0.5% yeast extract (Bacto™, BD Biosciences) and 0.05% NaCl (Sigma Aldrich, St Louis, MO, USA). The pH was adjusted to 7.4.

### 2.2. Growth Curves

Fresh overnight cultures of strains of interest were diluted 100-fold in fresh LB broth and incubated with vigorous aeration at 37 °C until *A*_600_ reached 0.48. The culture was then diluted 100-fold in prewarmed fresh broth and grown as before. Samples were taken every 30 min, diluted to 10^−7^ in M9 minimal medium without added glucose, and 10 μL aliquots of each dilution were immediately dropped onto LB agar plates. For each dilution series, two sets of drops were spotted. Colonies were counted after incubation for 18–24 h at 37 °C. Mean colony numbers from both spots were calculated and a growth curve plotted. A suitable period where growth was exponential was selected (usually between 30 and 180 min following dilution into fresh LB). For calculation of the doubling time, the LINEST function in Microsoft Excel (version 1808) was used to determine linear regression parameters for data points, which were calculated from averages per time point of seven independent experiments.

### 2.3. Genome Sequences

All *Escherichia coli* and *Shigella* genomic sequences were downloaded from the NCBI nucleotide database. Accession numbers for all genome sequences used in this study are included in Tables S2 and S3.

### 2.4. Alignment of ter Sequences

Concatenated FASTA files for the 17 *E. coli* genomes (Table S2) and 11 *Shigella* genomes (Table S3), respectively, were turned into indexes using BOWTIE2 processed with R (version 4.0.3) and the RBowtie2 package (version 1.10.1). FASTQ files containing the 10 primary *ter* sequences (*A–J*) were aligned against the index databases, relaxing the maximum penalty to 4 to get a hit for all *ter* sites. The resulting SAM files were converted into BAM format using the Rsamtools package (version 2.4.0) then cleaned using the Bioconductor package Biostrings (version 2.56.0) and dplyr (version 1.0.5). Using the start positions obtained from BOWTIE2, the Biostrings *subseq* function was used to extract genome-specific *ter* sequences. Finally, the sequence outputs were aligned with the DECIPHER (version 2.16.1) and MSA (version 1.20.1) packages utilising the Muscle alignment algorithm, which allowed filtering of illegitimate *ter* sites. For sequences to be recognised as legitimate, they needed to contain the crucial C6 base and have fewer than 5 mismatches compared with the MG1655 reference, while retaining inner core bases also known to be important for binding of Tus. Aligned *ter* site sequences were then visualised highlighting identical bases using the msaPrettyPrint function within MSA.

### 2.5. Architecture of the Termination Area across Phylogroups

The position of each *ter* site with strand information was contained in the Bowtie2 output file using the above method then cleaned with the dplyr, following the *ter* site filtering process. Cleaned dataframes containing sequence and position information were turned into dna_seg objects using the genoPlotR library (version 0.8.10) where shapes were specified based on an in-house function to show *ter* sites in either permissible or blocking orientations, with the triangles pointing in the permissible direction. To visualise the termination area in the context of other important genes, many individual dna_seg dataframes were concatenated to generate genome maps with overlapping gene information (e.g., tRNA) and other sites of interest (e.g., *matS*).

### 2.6. Sequence Alignment of Tus Proteins from All Phylogroups

Databases that included the genomes of interest were generated in the command line and the TBLASTN program was executed to align Tus from MG1655 against the genomes, with an E value specified as 1 × 10^−19^. Raw CSV outputs were read into R and cleaned using Biostrings to manipulate the proteomic data into alignment objects using the DECIPHER and MSA packages, as mentioned above. By using the msaPrettyPrint function once again, the Tus alignment figures were generated highlighting identical amino acid residues.

### 2.7. Detection of ter Sites within Coding Sequences

For the prediction of coding sequences, the Glimmer plugin [32] was used through Geneious Prime to annotate each genome that contained separate tracks of *ter* sequences. The *ter* sequences that showed overlap with a predicted gene were recorded in Excel.

### 2.8. Availability of Scripts Used in This Study

All scripts used for the analyses described above are available in the GitHub repository and can be accessed here: https://github.com/DanielGoodall/Goodall_etal2021.

## 3. Results

### 3.1. A Functional Replication Fork Trap Might Provide a Growth Advantage in E. coli

While in *E. coli* the initial analysis of cells lacking Tus protein did not reveal any major phenotypes [25,26], clear effects of the absence of Tus were subsequently reported [16,17]. Notably, the absence of Tus protein can result in low but detectable levels of chromosomal over-replication [33], an effect also observed in high-resolution replication profiles generated via WGS [23] (Figure S1). Over-replication of the termination area can be significantly exacerbated by additional mutations in PolI, RecG or 3′ exonucleases [23,33,34,35,36,37]. Moreover, the over-replication observed in these particular mutants is not a consequence of lacking Tus, as it can be observed in the presence of Tus [23,34,35,36,37].

In our recent work with strains containing multiple ectopic replication origins, we observed that *Δtus* single mutants consistently showed a mild elongation of the doubling time by between 1 and 2 minutes in LB broth [20,38]. This longer doubling time might indicate that cells lacking Tus have a mildly reduced fitness, as recently suggested [24]. However, the presence of an ectopic replication origin distorts the normal replichore arrangement. Because the ectopic origin is closer to the replication fork trap, one of the replication forks is arrested every cell cycle [3,20,38]. Thus, the mildly elongated doubling time could be caused by forks stalled at *ter*/Tus complexes requiring additional processing steps, rather than the presence or absence of Tus. Cells with a gross asymmetry of replichore lengths were shown to require the RecA main recombinase for survival [39], in line with the observed formation of double-strand breaks at ectopically inserted *ter* sites [18].

To avoid the complication of additional origins, we measured the doubling time of MG1655 versus a *∆tus* single mutant. As before, we found an elongation of the doubling time. However, the effect is less pronounced than in our constructs with additional ectopic origins (compare doubling times in Table 1). However, even though we saw some variation in the actual doubling times on different days, which lead to a relatively large standard deviation, our *Δtus* strain always grew more slowly than the Tus^+^ control run in parallel in all seven experiments, and a Wilcoxon signed-rank test confirmed statistical significance for *p* = 0.05. Thus, *Δtus* cells have a very subtle growth defect, which becomes more apparent if the replichore arrangement becomes skewed (Table 1) [20,38].

### 3.2. The Number and Distribution of ter Sites Is Similar in All E. coli Phylogroups

*E. coli* can be found in the gastrointestinal tract of a variety of warm-blooded animals, and host populations are strongly shaped by both factors specific to the host as well as the environment [40]. Consequently, *E. coli* populations can be quite diverse. Comparison of an increasing number of fully sequenced *E. coli* genomes revealed a core of approximately 2200 genes which are conserved in all isolates [41]. However, the systematic analysis of a larger number of strains revealed a pan-genome of more than 13,000 genes [41].

*E. coli* strains have been classified, revealing a variety of phylogenetic groups [42], the most prominent of which are A, B1, B2, D, E and S [43,44]. Commensal strains in humans belong mostly to phylogroup A [40], while pathogenic strains belong mostly to phylogroups B2 and D [44,45]. The enterohemorrhagic O157:H7 strains are in group E, whereas *Shigella* falls into the additional group S [46]. In a recent analysis, over 100,000 *E. coli* genomes were used for an extensive phylogenetic analysis [42]. This analysis allowed the more detailed definition of 14 phylogroups G, B2-1, B2-2, F, D1, D2, D3, E2, E1, A, C, B1, Shig1 and Shig2. The phylogenetic analysis showed an early separation of phylogroup D (D1–3), followed by a separation of phylogroups F, G, B2-1 and B2-2 from the remaining groups. Eventually phylogroups E, A, S, C and B1 separated [42]. The analysis of gene gain, gene loss and gene duplication ratios showed significantly different gain/loss/duplication ratios of all phylogroups, even between phylogroups that are thought to share an ancestral history, highlighting the significant divergence across the different phylogroups [42]. In this study, we restricted our analysis to the phylogroups A, B1, B2, D, E and S. Despite slightly less differentiation, our analysis still covers the divergence across the entire phylogenetic tree [42].

In *E. coli* K12, which belongs to phylogroup A, the innermost *ter* sites *A*, *B*, *C*, *D* and *E* are not part of open reading frames, while the outer *ter* sites *F*, *G*, *H*, *I* and *J* are [15]. Thus, *ter* sites *F–J* might be conserved mainly due to the conservation of the genes they are part of [15]. Nevertheless, the variability of *ter* sites *A–E* should give us an indication of how much they contribute to the fitness in *E. coli.* The very mild effect on the doubling time (Table 1) might suggest that the absence of a fork trap is of little consequence. In this case, we would expect to see increased variability of the sequence and location of these *ter* sites, as such variability would come at little cost.

**Figure 2:**
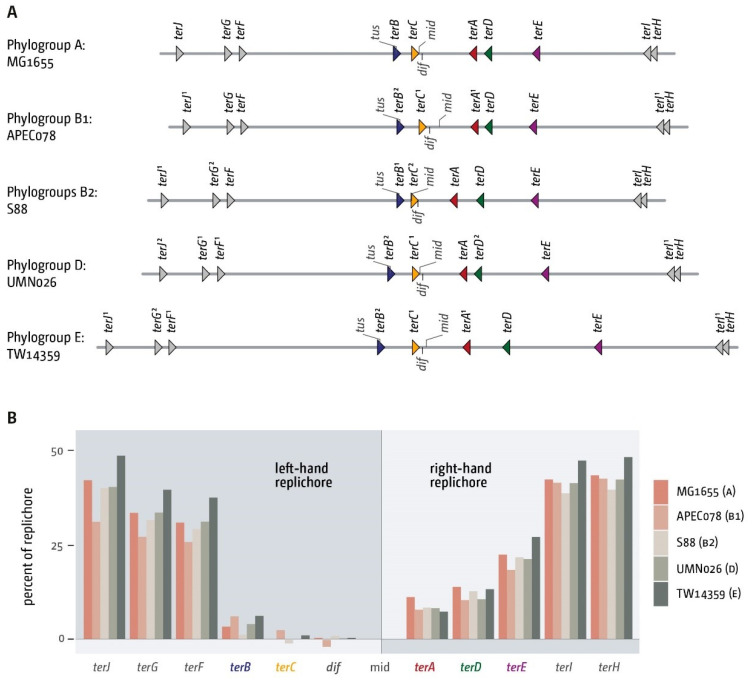
Architecture of the replication fork trap in genomes from the five *E. coli* genomes representing the phylogroups A, B1, B2, D and E. (**A**) Absolute positions of all primary *ter* sites in five *E. coli* genomes representing the phylogroups A, B1, B2, D and E. All *ter* sites are identified by triangles. The chromosome dimer resolution site *dif* is indicated by a tick mark. Orientation of the *ter* sites is indicated by triangle orientation. Forks meeting the tip of the triangle first will be blocked. If single nucleotide changes occur in comparison to the MG1655 reference genome, the number of changes is shown in superscript. The strand location of *dif* is indicated by the direction of the marker. The marker pointing downwards indicates that the *dif* sequence as defined in [48] is on the (−)-strand. (**B**) Comparative analysis of the relative positions of *ter* sites within the chromosomal context in five *E. coli* genomes representing the phylogroups A, B1, B2, D and E. To compensate for the different genome sizes the theoretical midpoint of each genome was calculated and used as basis for the analysis. The distance of all *ter* sites, as well as the chromosome dimer resolution site *dif*, were calculated as percent of the replichore (so *oriC* would be 100%). This gives an indication of how much the relative position of these elements varies between the genomes. Phylogroups are indicated in brackets after the strain name.

We started with the analysis of a single genome for each of the phylogroups B1, B2, D and E, with MG1655 (A) as a reference (Figure 2). This initial analysis revealed some variability of genome sizes (Table 2), but all *ter* sites could be identified (Figure 2A). As in MG1655, the innermost *ter* sites *A*, *B*, *C* and *D* are present and form a relatively small termination area which spans between 4% and 6% of the chromosome, as observed in MG1655 (Table 2). To take the different genome sizes into consideration, we calculated the arithmetic mid-point of each chromosome, based on *oriC*, and calculated the positions of *ter* sites as percentage of the replichore (Figure 2B). The analysis revealed that for four of the five genomes investigated, the arithmetic mid-point is located between the innermost *ter* sites, in close proximity to the chromosome dimer resolution site *dif*. Only in one strain, S88, was the arithmetic mid-point located just outside of *terC*, between *ter* sites *C* and *B* and therefore outside of the innermost termination area. However, as forks in *E. coli* do not proceed absolutely symmetrically [47], it is not clear without experimental work whether forks regularly get arrested by *terC* in S88. The analysis revealed some variations in the relative positions of some *ter* sites, but especially the relative location of the innermost *ter* sites is quite similar (Figure 2B).

**Figure 3:**
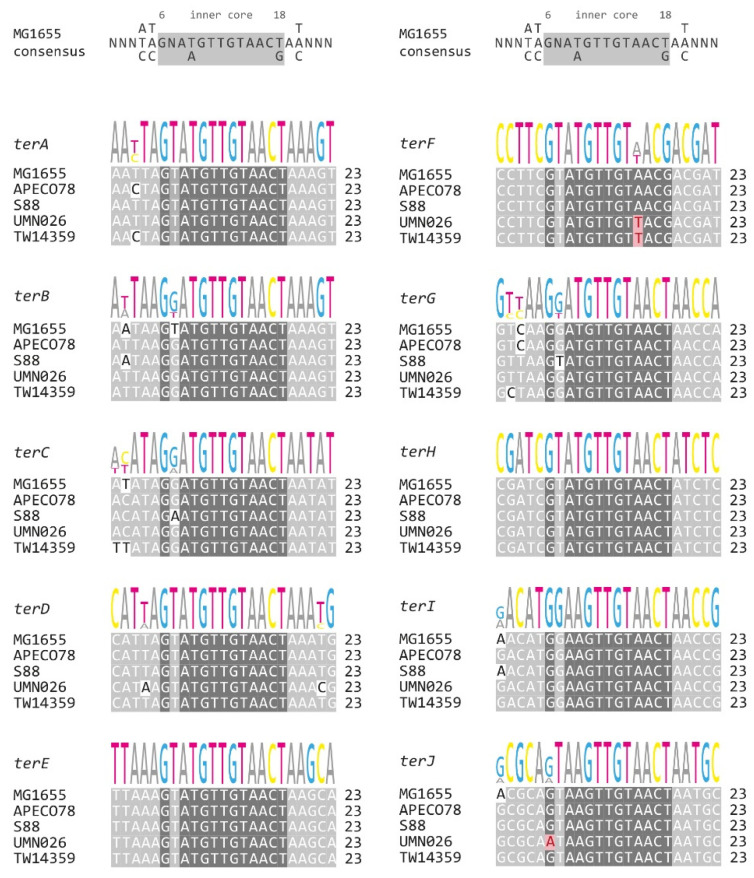
Sequence comparison of all *ter* sites from the 5 phylogroups in comparison to the MG1655 sequences. The *ter* consensus sequence derived from MG1655 is shown at the top. The phylogroups shown are A (MG1655), B1 (APEC078), B2 (S88), D (UMN026) and E (TW14359). Base pairs 1–5, 7 and 19–23 show a high degree of variability and are shaded in a lighter grey. The highly conserved core (6, 8–18) is highlighted by a darker grey. Mismatches unlikely to interfere significantly with binding have a white background shading. Mismatches within the core conserved sequence that are likely to interfere with binding are highlighted in red. Relative nucleotide frequencies are shown above as scaled letters.

**Figure 4:**
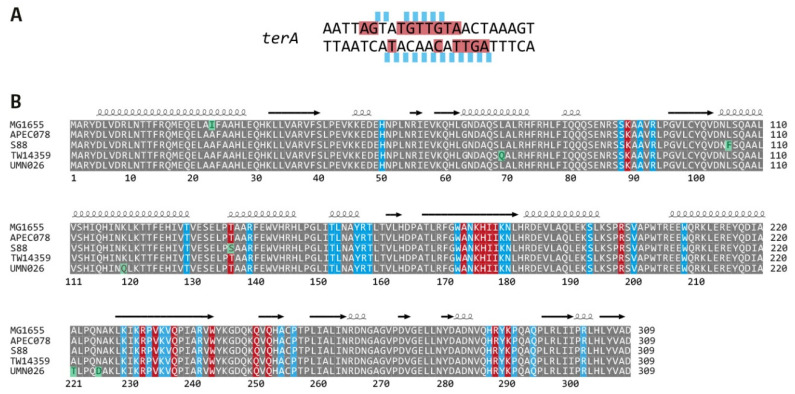
Interactions between Tus protein and *ter* sequences. (**A**) *terA* sequence in which interactions of Tus protein with the DNA backbone or individual bases is highlighted, as previously described [49]. Backbone contacts are shown in light blue above the *terA* sequence, where bases in direct contact with Tus are highlighted in red. (**B**) Sequence alignment of *E. coli* Tus proteins from the 5 phylogenetic groups. Identical residues are shaded grey, whereas any individual changes are highlighted in green. Those residues that interact with the DNA backbone of the *terA* sequence are highlighted by a blue block, while residues that make sequence-specific contacts with the *terA* DNA are highlighted with a red block, as described in [49]. Secondary structure information provided above is based on analysis using the ENDscript 2 server [54].

Next, we analysed the sequence variations of all primary *ter* sites. For some *ter* sites, we observed sequence variabilities of 1 or 2 bp. However, variations were mostly restricted to the outer sections of the *ter* sequences (Figure 3), which are not directly in contact with the Tus protein (Figure 4A) [16,49]. Some variability was also observed for position 7, but while lying in the core *ter* sequence, it has been shown before that this position can be changed, with little effect on the stability of the protein-DNA interaction, as it does not make any base specific contacts with Tus in either the bound or locked structure [50].

There were only three changes that are likely to reduce the stability of the protein-DNA complex. G6 of *terJ* in UMN026 showed a G to A transition mutation (Figure 3), which is likely to substantially reduce stability of the *ter*/Tus complex and therefore the ability to block replisome progression [16,50]. Similarly, *terF* showed an A to T transversion at position 15 both in UMN026 and TW14359 (Figure 3). This inner core region interacts significantly with Tus protein and any change introduced reduced Tus binding [50]. Thus, it seems likely that *terF* has a reduced binding affinity in these two backgrounds. However, both *terJ* and *terF* are a considerable distance from the fork fusion area, and it is not clear how often replisomes will be blocked here in normally growing cells [15].

In the five genomes analysed, we found that almost 90% of all variations (18 out of 21 SNPs total) occurred within the outer base pairs 1 to 5 and 19 to 23, which have little or no interaction with Tus protein based on the crystal structure [16,49], or at the variable position 7. More than half (11 out of 21) of these changes were observed in the four innermost *ter* sites, which are not part of open reading frames, while some of the outer *ter* sites (*terE* and *terH*) showed no changes whatsoever. This high degree of similarity of *ter* sites *F* to *J* is likely to be influenced by the need to maintain the genes these *ter* sites are part of. In contrast, this particular selection pressure does not apply for the innermost *ter* sites, explaining why for these *ter* sites, especially the outer sequences that do not interact directly with Tus, can be quite variable.

To establish that we had not selected unrepresentative members of the phylogroups in our initial analysis, we did an in-group comparison of the *ter* site patterns of three members of phylogroups A, B1, B2, and E, as well as five for phylogroup D (Figure S2), based on the recent phylogenetic analysis of these groups [42,46,51,52]. For phylogroups A, B1, B2 and E we again found variations of the overall genome size and proportion covered by the fork trap, but the order of *ter* sites appeared identical in all genomes analysed (Figure S2). Only in strain EDL933 from phylogroup E we observed what appears to be an inversion of the inner termination area, spanning *terA*, *terC* and *dif*. However, as two *ter* sites with opposite orientation are flipped, this inversion will not alter the functionality of the fork trap in this strain. Forks will still be forced to fuse in between *terA* and *terC* in EDL933.

Within phylogroup D, we found more variability. Three of the genomes analysed had a pattern of *ter* sites that matched the pattern observed in all other strains. However, two particular genomes differed. SMS-3-5 appears to have a large inversion of the termination area, which spans *ter* sites *E*, *D*, *A*, *C* and *B*. As for EDL933, this inversion will not interfere with the functionality of the fork trap: replication will still be blocked between *ter* sites A and C, as both of them are inverted. The only change from this pattern was observed in IAI39, in which *ter* sites *A*, *D* and *C* are, in terms of orientation and relative location, as in MG1655, while *ter* sites *B* and *E* are swapped. In MG1655, *terB* overlaps the promoter of the *tus* gene [25] and in IAI39, we found indeed both *terB* and *tus* in the new location. IAI39 is the only genome where we do not see an inner termination area that is flanked by a relatively tight cluster of two *ter* sites. The *ter* sites *D*, *A*, *C* and *E* will still act as a fork trap (Figure S2), but *terC* and *E* are much further apart than *terC* and *B* normally are.

### 3.3. Functionality of the Fork Trap in the Phylogroups

We have not established experimentally whether the *ter* sites identified in the various *E. coli* strains are functional. However, the sequence comparison of all *ter* sequences (Figure S3) highlights that the vast majority of SNPs found are in the outer variable *ter* region. The crystal structure of *E. coli* Tus protein [49] shows that the main interactions are with the inner core of the *ter* sequence. The outer sequences are not resolved in the structure and will contribute little to binding, both in bound and in the locked structure [53], explaining the higher degree of variability observed.

We also analysed Tus sequence alignments from the five *E. coli* phylogroups (Figure 4). Tus is 309 amino acids long and highly conserved—we observed a total of seven changes across the five phylogroups (Figure 4B), with individual strains having between one and three changed positions. Six of the seven changes observed are solvent exposed. Only one variant, Thr/Ser at position 136 in S88, is a residue close to the *ter* binding site. This residue makes minimal contacts with the DNA backbone (at position 10) and is a highly conservative variation, which is unlikely to affect binding. Thus, the Tus proteins from all five phylogroups are highly likely to form fully functional *ter*/Tus complexes. Given that the core of all *ter* sequences is unchanged and Tus is likely to be fully functional, we conclude that all strains contain a fully active replication fork trap, with only some minor variations.

### 3.4. Analysis of Secondary ter Sites

One key question is how much variability would be expected for sequence elements such as *ter* sites. To investigate this question, we analysed whether the order of other chromosomal markers is conserved. We analysed the presence and locations of *rrn* operons, genes coding for ribosomal proteins, tRNA genes and aminoacyl-tRNA synthetase genes before [5]. tRNA and aminoacyl-tRNA synthetase genes are particularly evenly distributed throughout the MG1655 chromosome [5], and we were interested to see how much variability is observed in the order of these genes.

We found that the order of genes shows, with few exceptions, very little variation across the phylogroups (Figure S4 for an overview, Figure S5 for some details), suggesting that the conserved order of the *ter* sites might not be very informative.

However, plotting these genes revealed their absence from the inner termination area. For example, aminoacyl-tRNA synthetase genes are relatively evenly spread, with the exception of three broad gaps with a reduced density (shown by light grey areas for MG1655 in Figure S4). These gaps coincide with (a) the area where *rrn* operons *C*, *A*, *B* and *E* are located, (b) the location of a cluster of genes encoding for ribosomal proteins and (c) the termination area (Figure S4). Analysis of all *matS* sites, which bind MatP and define the Ter domain of the chromosome, are specifically located in the termination area, but some overlap between *matS* sites and aminoacyl-tRNA synthetase genes is evident. *ter* sites appear to be a more defining factor: the density of these particular genes is very low between the innermost *ter* sites *B*, *C*, *A* and *D*, and none are located in between the innermost *ter* sites *C* and *A* (Figure S4).

The conclusion that the order of essential genes shows little variability even between the distant phylogroups fits with the observation that bacterial genome plasticity is often found in defined regions (“Regions of Genome Plasticity”, RGPs), such as pathogenicity clusters, prophages and regions encoding recombinases [55]. Indeed, for prophages in the *E. coli* genome, the mutational decay was reported to be high [56]. Deletion rates of materials not actively selected for were also shown to be high, leading to the typical compact bacterial genomes with a low number of pseudogenes [57]. From this perspective, the similar order of all primary *ter* sites is not too surprising. However, the consistent presence of the inner *ter* sites, which are outside of open reading frames, suggests that they have an important physiological role.

As the order of genes offers relatively little insight, we took two different approaches. Firstly, we included 25 bp upstream and downstream of all *ter* sequences in our sequence alignments (Figure S6). If it is specifically the binding of Tus that drives conservation of *ter* sites, we would expect for the *ter* sites outside of ORFs to see a relatively high degree of variability of the sequences surrounding the *ter* site, while variation of the actual *ter* site should be lower. This is indeed the case in the first approximation, as for example *ter* sites *A* and *D* have a much higher variability of the surrounding sequences than *ter* sites *F* to *J*, which all show a high degree of similarity (Figure S6). However, the analysis comes with some caveats. For *terB,* a relatively high degree of similarity is observed, but *terB* is located in the promoter of the *tus* gene [25], which will result in a higher degree of similarity. We were surprised to find a high degree of sequence similarity for *terE* (Figure S6). *terE* is not part of the innermost *ter* sites on most genomes analysed, and for that reason it is not very surprising that arrested forks could not be detected at *terE* in wild type cells [15]. However, *terE* is not part of known or predicted ORFs (Table S4), and yet *terE* itself and the surrounding sequences are highly similar. It will be interesting to analyse this particular region in the future to establish whether the sequences might be conserved due to an unpredicted ORF or other functional elements.

Secondly, we analysed the variability of the secondary *ter* sites *K*, *L*, *Y* and *Z*. The *ter* sites *K* and *L* are in the same replichore as *ter* sites *C*, *B*, *F*, *G* and *J* and share their direction (Figure 1A), but both have very low fork arresting activity [15]. *ter* sites *Y* and *Z* are both origin-proximal and in blocking orientation for forks coming from *oriC* (Figure 1A). Fork blocking activity is, again, very low unless *tus* is over-expressed [15]. *ter* sites *K*, *L* and *Z* are part of open reading frames in MG1655 [15], whereas *terY* is not. Thus, the analysis especially of *terY* might give us a better idea of the expected level of variability for a relatively short sequence with low or no selection pressure at the sequence level.

**Figure 5:**
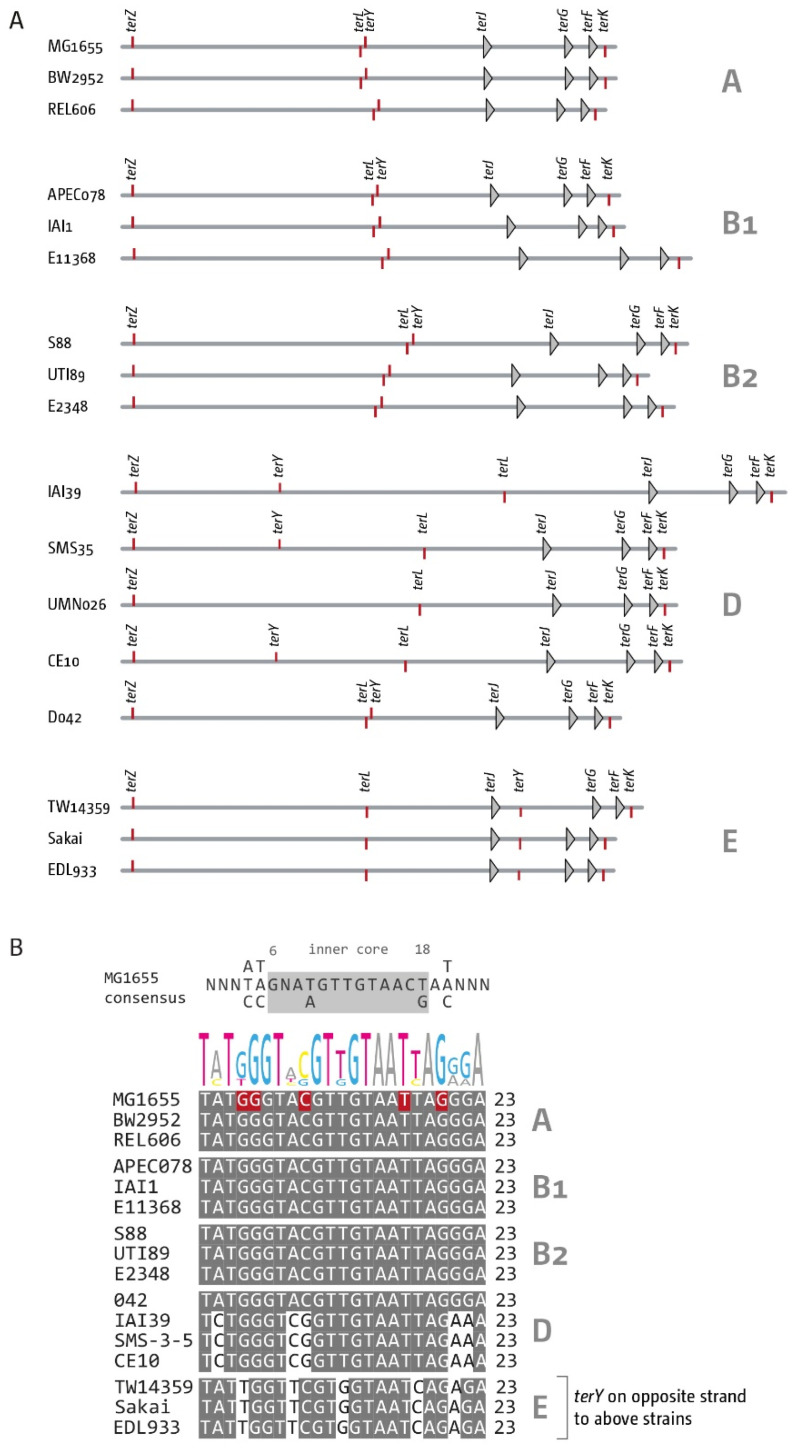
Distribution and sequence comparison of the secondary *ter* sites *K*, *L*, *Y* and *Z* in *E. coli*. (**A**) Location and distribution of the secondary *ter* sites *K*, *L*, *Y* and *Z* in phylogroups A, B1, B2, D and E. Genomes from the same phylogroups are clustered, with the group indicated on the right-hand side, and for groups A, B1, B2 and D, labels are only shown at the top. For phylogroup D, labels are shown for each genome because of the higher variability. Primary *ter* sites *F*, *G* and *J* are shown for orientation purposes (compare with Figure 1A). All primary *ter* sites are identified by triangles. Because some of the secondary *ter* sites are very close to each other, secondary *ter* sites are shown by a red tick mark. If the tick mark is below the genome line forks approaching from the left will encounter *ter*/Tus complexes in the permissive orientation. If the tick mark is above the genome line, then forks approaching from the left will encounter *ter*/Tus complexes in the restrictive orientation. (**B**) Sequence comparison of all *terY* sites from all strains across all 5 phylogroups in comparison to the sequence found MG1655 sequences. The *ter* consensus sequence derived from MG1655 is shown at the top. In MG1655, 5 base pairs of *terY* differ to the *ter* consensus sequence, 2 of which are situated in the inner core sequence, which explains the low fork block activity observed [15]. These positions are highlighted in red for MG1655. All mismatches to the *terY* sequence in MG1655 occurring in the other genomes analysed are shown by a white background shading. Relative nucleotide frequencies are shown above as scaled letters. The relevant phylogroups are again indicated on the right-hand side.

As might be expected, given their associations with ORFs, *ter* sites *K*, *L* and *Z* were found in all the analysed genomes (Figure 5A). The presence of *terY*, however, was more variable. Out of the 17 genomes analysed, we were able to find it in 10 by using the same parameters as for the primary *ter* sites. When we allowed for five mismatches, we were able to find it in all but one genome (UMN026). The strains from phylogroups A, B1 and B2 all showed little sequence variation. However, considerable differences were observed in strains from phylogroups D and E (Figure 5). *terY* already shows some changes in the core sequence in comparison to the *ter* consensus sequence (highlighted in red in Figure 5), explaining the low fork blocking activity of *terY* [15]. In some strains within phylogroups D and E, we found a number of additional sequence changes, which were not restricted to the outer parts of the *ter* sites but occurred through the core as well. It is noteworthy that *E. coli* strain 042, which is part of phylogroup D, shows a sequence identical to MG1655 (Figure 5), showing that there is not absolute consistency across phylogroups.

The variability observed supports the idea that, without selection pressure, *ter* sites outside of open reading frames would be expected to be far more variable or become lost from the genome. The strict conservation of the core sequences of *ter* sites *A–E* supports the idea that these *ter* sites indeed play an important role. However, the consistent presence of the secondary *ter* sites *K*, *L* and *Z*, which have only an extremely weak fork blocking activity, also emphasises that *ter* sites that are part of ORFs are likely to be pseudo *ter* sites that are retained via the selection pressure for the associated genes, as suggested [15].

### 3.5. The Replication Fork Trap in Shigellae

Similar to *E. coli*, Shigellae can cause a wide range of illnesses in human and non-human primates. Shigellae are facultatively anaerobic, Gram-negative, rod-shaped bacteria that are non-motile [58]. Early studies based on population genetics have shown that Shigella strains fall within the *E. coli* clade [59]. A more detailed analysis has revealed that Shigella strains do not form a discrete group, but likely have arisen multiple independent times [27,28]. We therefore analysed the replication trap in *Shigella flexneri*, *S. boydii*, *S. sonnei* and *S. dysenteriae*, serotypes that were suggested to have arisen independently [28].

**Figure 6:**
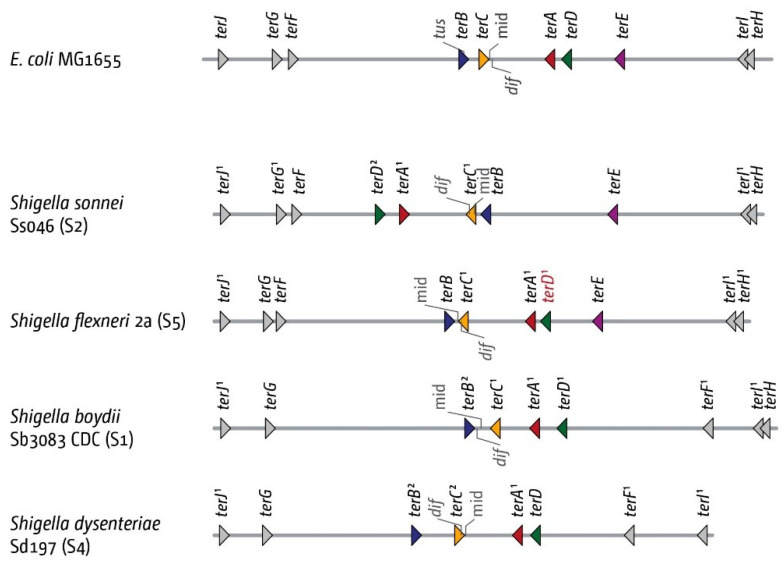
Architecture of the replication fork trap in genomes from four *Shigella* genomes representing the serotypes S1, S2, S4 and S5 (serotypes indicated in brackets behind the strain name). All *ter* sites identified, as well as the *dif* site, are indicated by markers. The orientation of the *ter* sites are indicated by the direction of the triangle (forks encountering the tip of the triangle would get blocked). The orientation of the *dif* chromosome dimer resolution site is indicated by the marker pointing upwards (indicating the (+)-strand) or downwards (indicating the (−)-strand). If nucleotide changes occur in comparison to the MG1655 reference genome, the number of changes is shown in superscript. *ter* sites with changes are key residues are shown in red.

**Figure 7:**
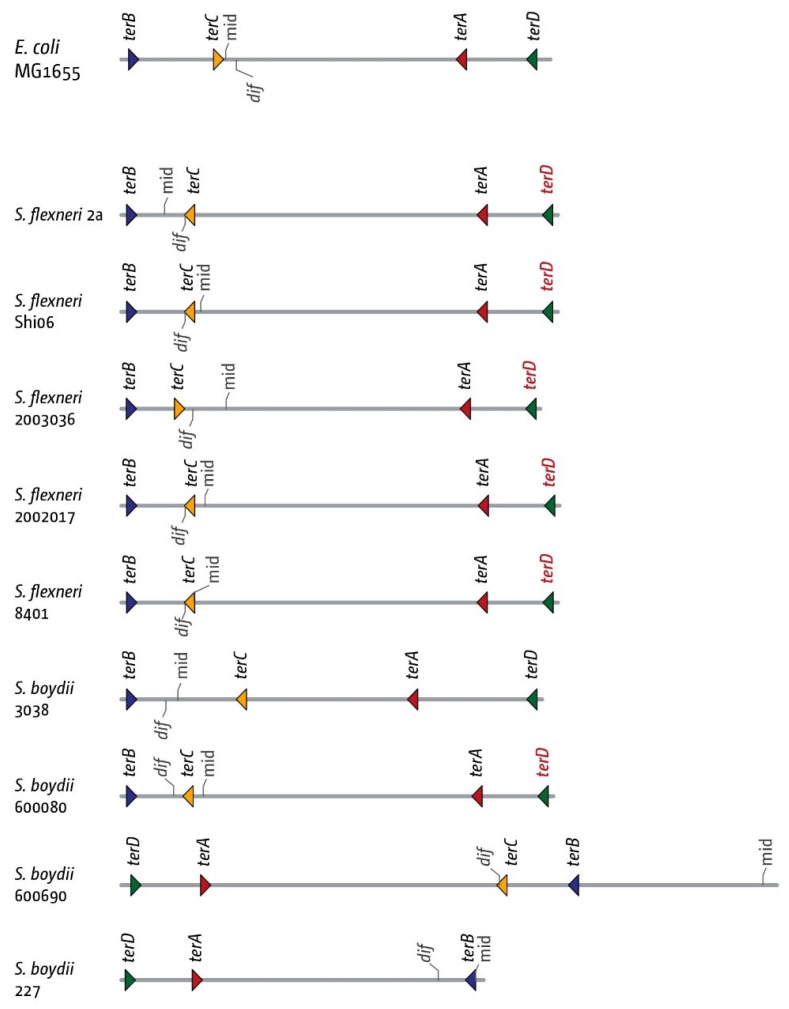
Details of the architecture of the inner replication fork trap in genomes from five *Shigella flexneri* (serotype S5) and four *Shigella boydii* (serotype S1) genomes. The inner *ter* sites *A*, *B*, *C* and *D* are identified, as well as the chromosome dimer resolution site *dif*. The orientation of the ter sites are indicated by the direction of the triangle (forks encountering the tip of the triangle would get blocked). The orientation of the *dif* chromosome dimer resolution site is indicated by the marker pointing upwards (indicating the (+)-strand) or downwards (indicating the (−)-strand). The *terD* sites highlighted in red indicate a change in the G6 residue, which will make the ability of this *ter* site to block a progressing replication fork much less efficient (see Figure S7). As the main focus of the comparison are the relative positions of *terA*, *terC*, *terB* and *dif*, we have not indicated the number of nucleotide changes in this figure.

We found the four innermost *ter* sites in our initial analysis without difficulty (Figure 6), with exception of S. boydii 227, where we could not identify *terC* (Figure 7). Overall, there appears to be a higher degree of variability. In *S. sonnei* Ss046, we found all 10 *ter* sites, but the inner termination area appears to be inverted. While the inversion changes the relative architecture of the termination area, it leaves the fork trap intact. In *S. flexneri,* we were also able to identify all 10 *ter* sites, but for the *terD* sequence, the highly conserved G6 is changed to an A (Figure S7), suggesting that Tus binding as well as blocking activity will be much reduced [50]. *terE* was missing from all *S. boydii* genomes we analysed, and *terF* appears to have moved into the opposite replichore, an effect also seen in *S. dysenteriae.* We have not further analysed whether this was based on a translocation event or a change in the *terE* sequence, which, in *E. coli*, would be located between *ter* sites *I* and *D*. *terH* also appears to be missing in *S. dysenteriae* (Figure 6). Despite the increased variability in the relative positions of the *ter* sites, we have no indication that the existing *ter* sites might be inactive in Shigellae, as their *ter* core match the MG1655 consensus sequence (Figure S7).

The analysis of the Shigella genomes revealed an additional feature. In both *S. boydii* 3083 and *S. flexneri* 2a, the *dif* chromosome dimer resolution site is located between *terC* and *B*, rather than *terA* and *C* (Figure 6). In all *E. coli* genomes analysed, *dif* is located in between the innermost *ter* sites *A* and *C*. Indeed, it was proposed that the majority of fork fusions might take place at chromosome dimer resolution site *dif*, because the inversion point of the chromosomal GC skew indicated that replication must frequently terminate at *dif* [60]. Following this suggestion, both computer simulation studies and marker frequency analysis have provided data arguing against *dif* as the main fork fusion point [20,23,61,62].

However, the changed location of *dif* in some Shigella strains adds an additional argument to the discussion. In both *S. boydii* 3083 and *S. flexneri* 2a, the orientation of *terC* is changed: forks are forced to fuse between *terB* and *C*, rather than *terA* and *C*, as in *E. coli*. Thus, even though the relative position of *dif* has changed, it remains in the innermost termination area where forks fuse (Figure 6).

Following this observation, we analysed the termination area in a total of five *S. flexneri* and four *S. boydii* genomes. Out of five *S. flexneri* genomes, we found one where the architecture of the fork trap reflects the one found in MG1655. In all others, *dif* is located between *ter* sites *C* and *B*, with *terC* having changed orientation so that forks are trapped in between *terC* and *terB*. This change is often associated with a *terD* site where G6 is changed to an A (Figure S7). For *S. boydii,* we found one genome where *dif* is located in between *terC* and *terA*, as in MG1655. For this strain, the entire inner termination area is inverted, which leaves the functionality unaffected. In two further strains, we found *dif* to be located between *terC* and *terB*, and, as for *S. flexneri*, *terC* had a changed orientation, forcing forks to be trapped between *terC* and *terB*. Finally, in one strain we were unable to find *terC* (Figure 7; *S. boydii* 227).

As both *dif* and *ter* sites are asymmetric, their relative position can be easily determined. If *terC* is notated as in Figure 3, and *dif* is notated as GGTGCCATAATGTATATTATGTTAAAT [48], then in MG1655 both are located on different strands. Thus, the situation observed could have arisen from a small inversion that spans both *terC* and *dif*. Indeed, for *S. boydii* 600080, we observed that both *terC* and *dif* are on opposite strands to MG1655 (Figure 7). However, for all other *S. boydii* and *S. flexneri* genomes where *dif* is located between *terC* and *terB*, we found that only the relative location of *terC* has changed, placing *terC* and *dif* on the same strand (Figure 7). Thus, a more complex change must have taken place to lead to this arrangement, which makes it tempting to speculate that not only the replication fork trap per se but also the relative location of *dif* within it are an important feature of chromosomal architecture.

### 3.6. The Replication Fork Trap in Salmonella and Klebsiella

Another important question is how the replication fork trap might be structured in other bacterial species. *tus*-related sequences have been found in most Enterobacteriales [24], and it has been shown before that the expression of *tus* genes from *Salmonella enterica*, *Klebsiella ozaenae* and *Yersinia pestis* in *E. coli* resulted in the formation of functional *ter*/Tus complexes, even though the blocking activity of the heterologous complexes were not as strong [25,63]. However, the sequences of Tus from *Salmonella*, *Klebsiella* and *Yersinia* show substantial differences, and without a biochemical analysis it is not possible to deduce whether sequence variations seen in potential *ter* sites might lead to inactivation or are based on different interactions with the species-specific Tus protein.

**Figure 8:**
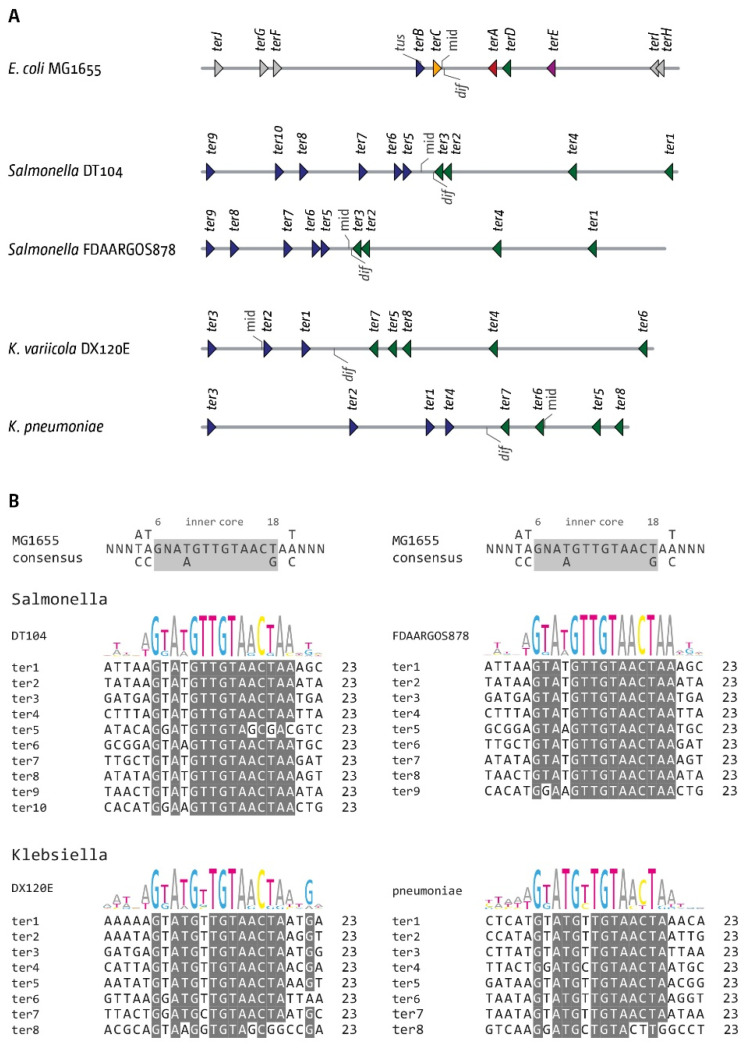
The replication fork trap in *Salmonella* and *Klebsiella* genomes. (**A**) Architecture of the replication fork trap in two *Salmonella enterica* genomes, as well as *Klebsiella variicola* and *K. pneumoniae*. The organisation of the *E. coli ter* sites is shown at the top as reference. All *ter* sites identified, as well as the chromosome dimer resolution site *dif*, are indicated by markers. The orientation of the *ter* sites are indicated by the direction of the triangle (forks encountering the tip of the triangle would get blocked). All *ter* sites identified were numbered, as the relationship to the *ter* sites in *E. coli* is not clear. (**B**) Sequence comparison of all *ter* sites from the two *Salmonella enterica*, the *Klebsiella variicola* and the *K. pneumoniae* genomes. The *ter* consensus sequence derived from MG1655 is shown at the top for reference purposes. All base pairs that are identical are shown by a grey background.

To get at least some insight into the structure of fork traps in other species, we investigated two *Salmonella enterica* genomes, as well as *Klebsiella variicola* and *Klebsiella pneumoniae* genomes. To identify potential *ter* sites, we used precisely the same parameters as for *Shigella*, allowing a maximum of five mismatches. For all genomes, we excluded *ter* sites that had a number of mismatches throughout and, in addition, missed the critical G at position 6. All other potential *ter* sites found were included (Figure 8A). Given that a significant number of mismatches were observed in all putative *ter* sites (Figure 8B), we numbered them, rather than using the nomenclature used in *E. coli*.

Without the biochemical characterisation that the *ter* sites found are indeed active, the analysis must remain limited. However, all genomes show a pattern of putative *ter* sites that is strikingly similar to what is observed in *E. coli*. In both *Salmonella* genomes, we found an area opposite to *oriC* that is flanked by a tight pair of clustered *ter* sites. The chromosomal mid-point and *dif* chromosome dimer resolution site both are located between the innermost *ter* sites. We noted that the pair of *ter* sites on the left-hand side in Figure 8A is followed by another *ter* site, a pattern reminiscent of *ter* sites *A*, *D* and *E* in *E. coli*. Similarly, both in *Klebsiella variicola* and *K. pneumoniae* we found that the *dif* chromosome dimer resolution site was flanked by at least two *ter* sites which would form a trap. Interestingly, in these genomes, we found that the arithmetic mid-point is not located within the innermost *ter* sites. If these *ter* sites were to form functional *ter*/Tus complexes, this would be in line with our hypothesis that keeping the *dif* chromosome dimer resolution site close to the fork fusion point is one of the important functions of the replication fork trap. It will be intriguing to investigate this point further in future studies.

## 4. Discussion

While the biochemical aspects of *ter*/Tus complexes have been studied extensively, we still know surprisingly little about the precise physiological function of the replication fork trap. *tus*-related sequences have been found in most Enterobacteriales, in the Pseudoalteromonas and in most Aeromonadales [24]. A fork trap system is also present in the Bacillales, which involves *ter* sequences and the terminator protein RTP [24]. However, no similarity is found between the *ter* sequences in *E. coli* and *B. subtilis* nor between Tus and RTP, suggesting that these fork trap systems have evolved via convergent evolution [16]. These findings suggest that fork trap systems have an important role in DNA replication. However, early studies did not report any phenotype of cells lacking Tus [25], and our own growth curves showed only a very mild growth delay of cells lacking Tus protein (Table 1). Delays are slightly more pronounced in cells in which one fork almost always gets blocked at a *ter*/Tus complex [20,38], suggesting perhaps that the fork trap might be of particular importance in cells in which individual forks proceed beyond the boundaries of the termination area. Indeed, in normal growing cells, the majority of fork fusions take place at the chromosomal midpoint, away from any *ter*/Tus complexes [3,15,22,23].

If *ter*/Tus complexes do not normally contribute to the fork fusion process, when do they block fork progression, and of which forks? There is no doubt that the delay of one fork coming from *oriC* will result in the other being arrested at *ter*/Tus complexes. In normally growing wild-type cells, this scenario can be easily detected in vivo within a population, even though the numbers of arrested forks are relatively low [15]. One of the early hypotheses about the fork trap was that it prevents forks from entering the opposite replichore if the second fork is delayed at an obstacle, thereby preventing head-on clashes between replication and ongoing transcription [64]. However, why would this require a replication fork trap in *E. coli* that spans ~45% of the entire chromosome [5,16,17]? As the outer *ter* sites all are part of open reading frames, Duggin and Bell have predicted that these are pseudo *ter* sites that are conserved by selection pressure for the genes they are part of, rather than their functionality as a fork trap [15]. Our analysis of the secondary *ter* sites supports this idea (Figure 5). While the secondary *ter* sites *K*, *L*, *Y* and *Z* all have a very low fork blocking activity [15], the three *ter* sites associated with open reading frames are conserved. In contrast, *terY* is far more variable, both in terms of location and its precise sequence (Figure 5). The finding that *ter* sites with a low fork blocking activity are quite conserved if they are associated with ORFs supports the idea that the outer *ter* sites *F–J* might indeed be pseudo *ter* sites, as suggested by [15]. Interestingly, in MG1655, *terE* is not part of any ORF, but our analysis has shown that both *terE* as well as the surrounding sequences are surprisingly similar in all genomes investigated (Figure S6). Given that in wild-type cells no stalled forks were observed at *terE* [15], it is tempting to speculate that *terE* might also be a pseudo-*ter* site, with other currently unknown functional elements in the *terE* area driving the high similarity. This would leave the innermost *ter* sites A–D as the active replication fork trap, in line with the measured fork blocking activities [15].

In Shigellae, the replication fork trap appears to be somewhat more variable than in *E. coli* (Figure 6 and Figure 7). However, Shigella strains do not form a discrete group within *E. coli*, but have arisen multiple independent times [27,28], which will contribute to the variability observed. Nevertheless, the inner termination area still shows a great similarity in all the genomes we analysed (c.f. Figure 2 and Figure 6).

It was shown before that any gross asymmetry in replichore lengths in *tus^+^* cells render the strain dependent on the RecA recombinase for viability [39], in line with results from ectopically inserted *ter* sites [18]. Such gross asymmetry also induced segregation defects [39]. These results suggest that a fork trap system might be involved in preventing the formation of major asymmetries in the length of the two replichores [39]. However, we also reported recently that when we engineered strains in which the native *ter*/Tus blocks got significantly in the way of DNA synthesis, the resulting strains were very quickly outgrown by cells carrying suppressor mutations, some of which showed gross chromosomal rearrangements (GCRs) that flipped all *ter* sites from restrictive to permissive orientations [3,38]. This demonstrates that if fork traps get significantly in the way of DNA synthesis, cells can lose these very quickly. In addition, our analysis shows that the innermost *ter* sites *B–E* were consistently outside of open reading frames (Table S4). If there was little or no selection pressure to keep these *ter* sites, a higher variability of all *ter* sites between the strains of the various phylogroups might be expected, precisely as observed for the secondary *ter* site *terY* (Figure 5), especially as the termination area in general and *ter* sites in particular are a recombination hotspot [65]. Some of the strains analysed show some gross chromosomal rearrangements in the termination area and elsewhere (SMS-3-5, IAI39; Figure S2), but these rearrangements only change the relative position of *ter* sites, while the fork trap itself was left intact (Figure S2).

Given that the fork trap is present in all strains investigated, are there additional clues that might clarify its function? In *E. coli,* a significant reported phenotype was in the context of R1 plasmid replication. For R1 replication, two forks are established, but one is arrested almost immediately and almost the entire plasmid is replicated unidirectionally by a single fork until it gets arrested as well at a single *ter*/Tus complex close to the plasmid origin [66]. Inactivation of this *ter*/Tus complex resulted in the accumulation of a variety of aberrant branched structures, including rolling circle replication intermediates and plasmid multimers [67]. A similar generation of complex structures was reported in vitro for a plasmid substrate, where functional *ter*/Tus complexes efficiently prevented over-replication and the formation of complex intermediates [68], a result recently confirmed in ‘replication chain reaction’ experiments [69]. Importantly, Krabbe and co-workers were able to show that generation of these intermediates interfered significantly with successful plasmid segregation [67]. Thus, the absence of a functional *ter*/Tus system triggers segregation defects and plasmid loss, which will impose a strong selection pressure.

Notably, Galli et al. found that the *tus* gene in the Pseudoalteromonadacae is systematically associated with the secondary chromosome, which is of plasmid origin. On this chromosome, the *tus* gene is always located next to the origin of replication [24]. The fact that in *E. coli terB* is located within the promoter region of *tus* [25] makes it tempting to speculate that the same is true for the Pseudoalteromonadacae, and the GC skew for the secondary chromosome in *Pseudoalteromonas haloplanktis* fits a unidirectional replication mode [70,71], suggesting that the mode of replication is similar to R1. If so, then the fork trap systems in R1 and the Pseudoalteromonadacae will be preventing the formation of aberrant termination intermediates that interfere with successful segregation.

The need for preventing the formation of fork fusion intermediates might have been the advantage when the fork trap system migrated into the *E. coli* chromosome. The absence of Tus results in mild over-replication of the chromosome [23,33] (Figure S1). This over-replication can be significantly exacerbated by additional mutations in PolI, RecG or 3′ exonucleases [23,33,34,35,36,37], suggesting that these enzymes are processing complex branched intermediates that form as a result of fork fusion events. In their absence, the intermediates persist for longer than normal, as suggested for R1 replication [3,5,23,34]. This hypothesis is supported by the observation that over-replication in cells lacking either RecG or 3′-exonucleases is significantly reduced following linearisation of the chromosome in the termination area, which prevents replication forks from fusing [5,23,72], and by the fact that over-replication can be induced in ectopic chromosomal locations in the absence of RecG if replication forks are forced to fuse here [37]. These observations are in line with the idea that intermediates formed as part of termination can trigger replication restart and lead to chromosomal over-replication. Importantly, this over-replication is normally contained within the termination area, where it can be rapidly processed, thereby leading to successful and accurate termination [34,72,73]. One important role of the fork trap, therefore, might be to prevent over-replication from duplicating extensive areas of the chromosome. This would involve forks that are not initiated at the origin, but are coming from other areas of the chromosome. Indeed, this hypothesis might also contribute to the explanation for our observation that the essential genes for RNA metabolism (Figure S4) are excluded from the termination area. If nucleolytic processing is taking place in this area on a regular basis to remove over-replication intermediates, the presence of essential genes would be expected to be quite dangerous. Any processing error that threatens the integrity of such a gene would have lethal consequences. Obviously, other effects, such as gene dosage, might also contribute and have been discussed [74].

**Figure 9:**
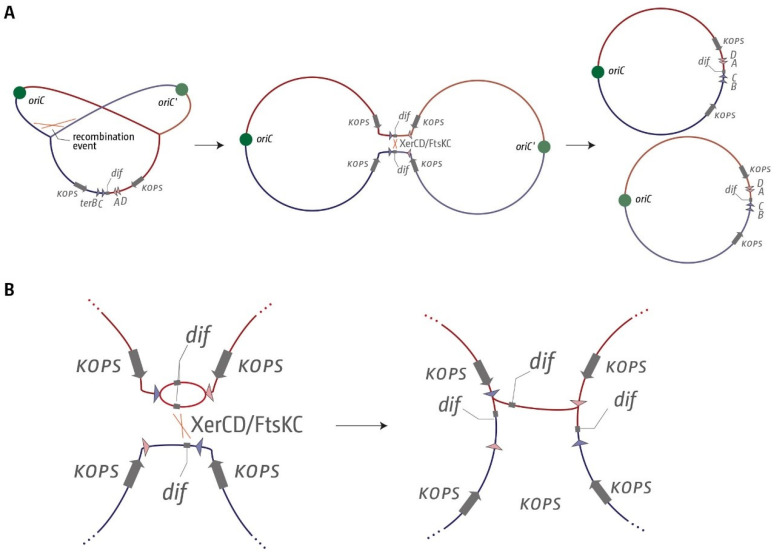
Dimer resolution of circular chromosomes. (**A**) Circular *E. coli* chromosome that is in the process of being replicated. Only the innermost *ter* sites *A*, *B*, *C* and *D* are shown. The general orientation of the KOPS sequences that allow directional travel of the FtsK protein are shown by grey arrows with the label ‘KOPS’. An odd number of recombination events can result in the formation of chromosomal dimers. If a chromosomal dimer is formed, the XerCD site-specific recombinase allows for a recombination event between the two *dif* sites, which results in the resolution of the chromosomal dimer. (**B**) If the termination area, including *dif*, is over-replicated in one of the two chromosomes, a recombination event between two of the *dif* sites will not result in full resolution of the chromosomal dimers. See main text for details.

Our findings in Shigellae might add another facet to the debate. We observed a correlation between a shift in the location of the chromosome dimer resolution site *dif* and a corresponding change in the precise location of the innermost termination area (Figure 6 and Figure 7). In order to successfully resolve chromosomal dimers in *E. coli*, the two *dif* have to be brought into close proximity, which then allows resolution by the site-specific recombinase XerCD (Figure 9A) [48]. This resolution process will work best if exactly two copies of *dif* are present, as otherwise there might be a chance of the wrong *dif* copies being used for resolution or, in case of an over-replicated termination area, a link between the chromosomes might remain (Figure 9B). Indeed, in *Vibrio cholerae,* the two existing *dif* sequences, while similar, show five different base pairs [48], which will prevent recombination between the two chromosomes. The availability of *dif* for dimer resolution also needs to be restricted. If two copies of *dif* were available before chromosome duplication is completed, unwanted resolution events might happen in the absence of a dimer. While computational analysis shows that *dif* and the natural fork termination point are not the same [61], the *dif* site is located in the termination area of the chromosome in the majority of bacterial chromosomes analysed [61]. In fact, in all genomes analysed in this study, *dif* was located inside the innermost *ter* sites, while the theoretical mid-point where forks would fuse if they moved at the same speed was far more variable (Figure 2, Figure 6, Figure 7, Figure 8 and Figure S2). Indeed, when we artificially moved *dif* outside of the termination area beyond *terB,* we found a significant elongation of the doubling time (Table S5). Thus, it appears that the arrangement of having the *dif* site located within the innermost *ter* sites ensures that two *dif* sites only become available for dimer resolution when the duplication process is essentially complete. These *dif* sites will only remain in close proximity for any length of time if chromosomes are dimerised. If chromosomes are successfully separated, segregation will move them apart. However, if chromosome over-replication in the termination area generates additional copies of *dif*, this will be problematic for chromosome dimer resolution unless the over-replication is contained and quickly processed (Figure 9).

The data available show that the purpose of the architecture of the replication fork trap in *E. coli* is unlikely to be the coordination of fork fusions. Instead, it is far more likely that one main function of the replication fork trap is to contain intermediates that can be formed as part of the final stages of DNA replication. Thus, it will prevent a fork coming from *oriC* from migrating out of the termination area, but it will also prevent progress of forks initiated elsewhere, including synthesis within the fork trap area as part of termination. The rapid processing of such intermediates will prevent a delay in full genome replication, but the removal of over-replicated sections will also allow control of the copy number of *dif*, ultimately leading to successful completion of DNA synthesis and chromosome segregation. Maintaining replichore directionality to avoid replication–transcription conflicts will indeed be a welcome side effect, but it is unlikely that it is the main driver for maintaining a fork trap area, especially as there is little indication that forks moving beyond the fork trap boundaries encounter major difficulties [20,38].

In addition, it was also proposed that *ter*/Tus complexes are involved in the coordination of chromosome segregation and cell division [24]. The protein MatP is specific for binding to the chromosomal Ter domain [8]. MatP not only connects the chromosome and the divisome, but also inhibits MukBEF activity, which contributes to forming an individual functional chromosomal domain [75]. A fork trap system will help to ensure that upon completion of DNA replication, the chromosomal Ter domain is positioned at mid-cell, as suggested [24]. Thus, it appears that the replication fork trap area might offer multiple interconnected benefits that contribute towards successful chromosome segregation.

## Figures and Tables

**Table 1:** Doubling times of various *E. coli* strains in the presence and absence of Tus.

Strain Background	Doubling Time (min)	SD	Reference
MG1655	19.1 ^a^	±1.3	This study
*∆tus*	19.6 ^a^	±1.2	This study
*oriC*^+^ *oriZ*^+^	20.6	±1.3	[20]
*oriC*^+^ *oriZ*^+^ *∆tus*	21.5	±0.7	[20]
*oriC*^+^ *oriX*^+^	22.3	±1.2	[38]
*oriC*^+^ *oriX*^+^ *∆tus*	23.1	±0.7	[38]

^a^—Doubling times are an average from seven independent experiments.

**Table 2:** Genome parameters of the different *E. coli* phylogroups.

Background	Phylogroup	Genome Size (Mbp)	Inner Termination Area (kb)	% of Total Genome Size
MG1655	A	4.641	267	5.76
APEC078	B1	4.798	243	5.05
S88	B2	5.032	190	3.77
UMN026	D	5.202	225	4.32
TW14359	E	5.528	238	4.31

## Data Availability

The genome data presented in this study are openly available in the NCBI nucleotide database. All relevant accession numbers are provided in Supplementary Tables S2 and S3. All scripts used in this study and described in Material & Methods can be accessed via Github (see Section 2.8). All other data are contained within this article and its Supplementary Material.

## Supplementary Materials

The following are available online at https://www.mdpi.com/article/10.3390/ijms22157928/s1, Figure S1: Replication termination in *E. coli* cells in the presence and absence of a functional replication fork trap, Figure S2: Architecture of the replication fork trap in genomes from groups of *E. coli* genomes representing the phylogenetic groups A, B1, B2, D and E, Figure S3: Sequence comparison of all *ter* sites across all phylogenetic groups from all *E. coli* genomes analysed, with the MG1655 sequences as reference, Figure S4: Location and distribution of genes and genetic markers involved in termination of DNA replication as well as translation, Figure S5: Location and relative order of aminoacyl tRNA synthetase genes within the chromosomes of the *E. coli* phylogroups A (MG1655), B1 (APEC078), B2 (S88), D (UMN026) and E (TW14359), Figure S6: Sequence comparison of all *ter* sites, including 25 bp upstream as well as downstream, across all phylogenetic groups from all *E. coli* genomes analysed, with the MG1655 sequences as reference, Figure S7: Sequence comparison of all ter sites across all serotypes from all *Shigella* genomes analysed, Table S1: *Escherichia coli* K12 strains, Table S2: List of all *E. coli* genomes used in this study, including NCBI nucleotide database access links, Table S3: List of all *Shigella*, *Salmonella* and *Klebsiella* genomes used in this study, including NCBI nucleotide database access links, Table S4: Location of the 10 major *ter* sites in relation to predicted open reading frames within the 5 main *E. coli* phylogenetic groups, Figure S5: Doubling times of *E. coli* strains with the chromosome dimer resolution site dif in its native or an ectopic location.
