# Supplementary material for: A Fork Trap in the Chromosomal Termination Area Is Highly Conserved across All Escherichia coli Phylogenetic Groups
Source: Int J Mol Sci. 2021 Jul 25;22(15):7928. doi: 10.3390/ijms22157928 (PMC8347550; doi:10.3390/ijms22157928)
Supplement: Supplementary file 1 [file ijms-22-07928-s001.zip › ijms-1292787-supplementary.pdf]

Supplementary Material

A fork trap in the chromosomal termination area is highly conserved across all Escherichia coli phylogenetic groups

Daniel J. Goodall[1], Katie H. Jameson[2], Michelle Hawkins[2] and Christian J. Rudolph[1,*]

*Corresponding author: christian.rudolph@brunel.ac.uk

1 Division of Biosciences, College of Health, Medicine and Life Sciences,
Brunel University London, Uxbridge, UB8 3PH, UK

2 Department of Biology, University of York,
Wentworth Way, York, YO10 5DD, UK

SUPPLEMENTARY FIGURES AND LEGENDS

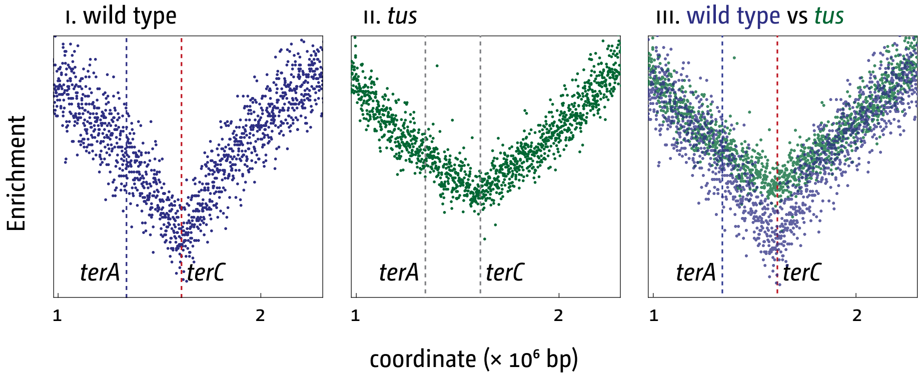

Figure S1. Replication termination in *E. coli* cells in the presence and absence of a functional replication fork trap. Shown is the marker frequency analysis of the fork fusion area of *E. coli* cells in which the replication fork trap in the termination area was inactivated by deletion of the *tus* gene. The number of reads (normalised against reads for a stationary phase wild type control) is plotted against the chromosomal location. The innermost *ter* sites *A* and *C* are shown by dotted lines. Individual profiles of MG1655 (blue) and *Δtus* (green) are shown in panels i and ii, while an overlay of both is shown in panel iii. The data were re-plotted from [2].

Phylogroup A

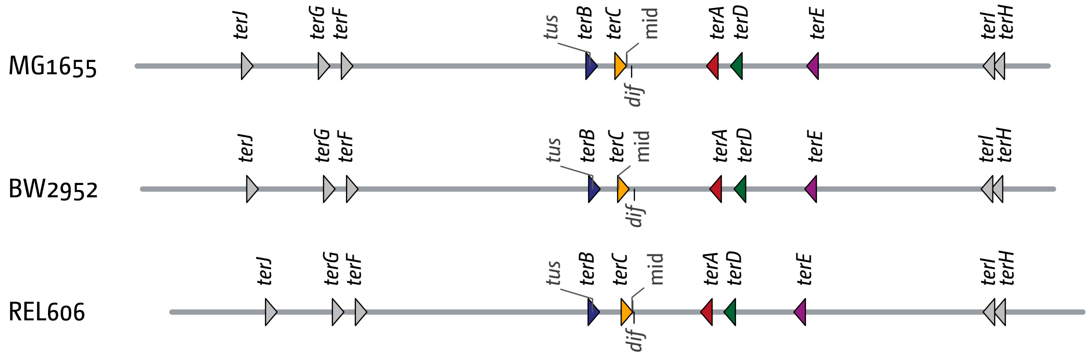

Figure S2. Architecture of the replication fork trap in genomes from groups of *E. coli* genomes representing the phylogenetic groups A, B1, B2, D and E. Orientation of the *ter* sites is indicated by triangle orientation. Forks meeting the tip of the triangle first will be blocked. If single nucleotide changes occur in comparison to the MG1655 reference genome, the number of changes is shown in superscript. The strand location of *dif* is indicated by the direction of the marker. The marker pointing downwards indicates that the *dif* sequence as defined in [4] is on the (–)-strand.

Phylogroup B1

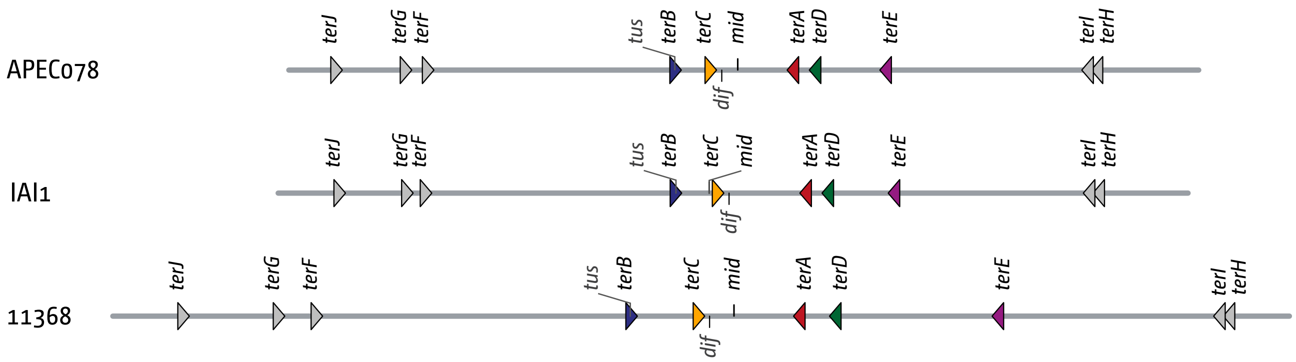

Figure S2 continued.

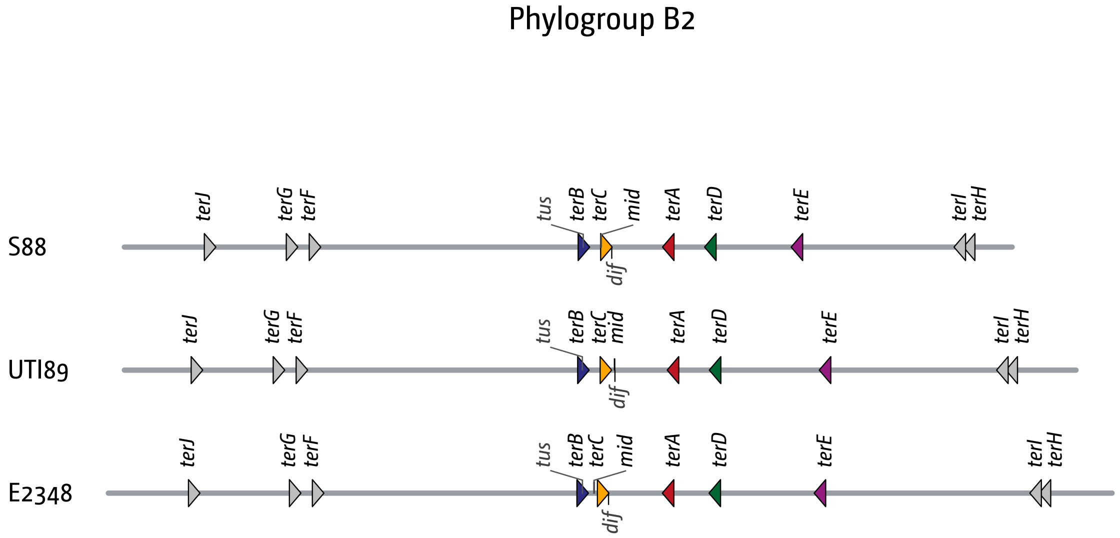

Figure S2 continued.

Phylogroup D

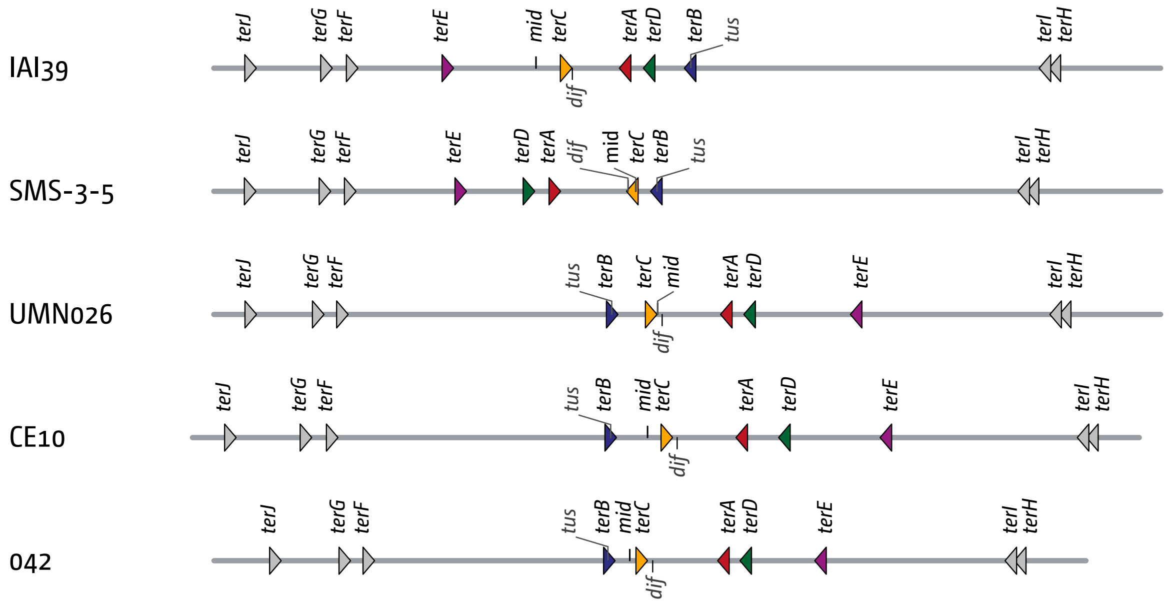

Figure S2 continued.

Phylogroup E

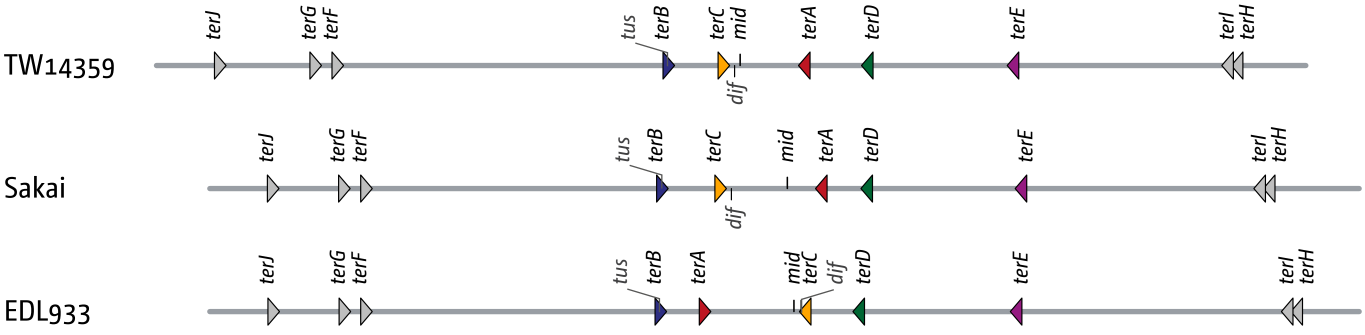

Figure S2 continued.

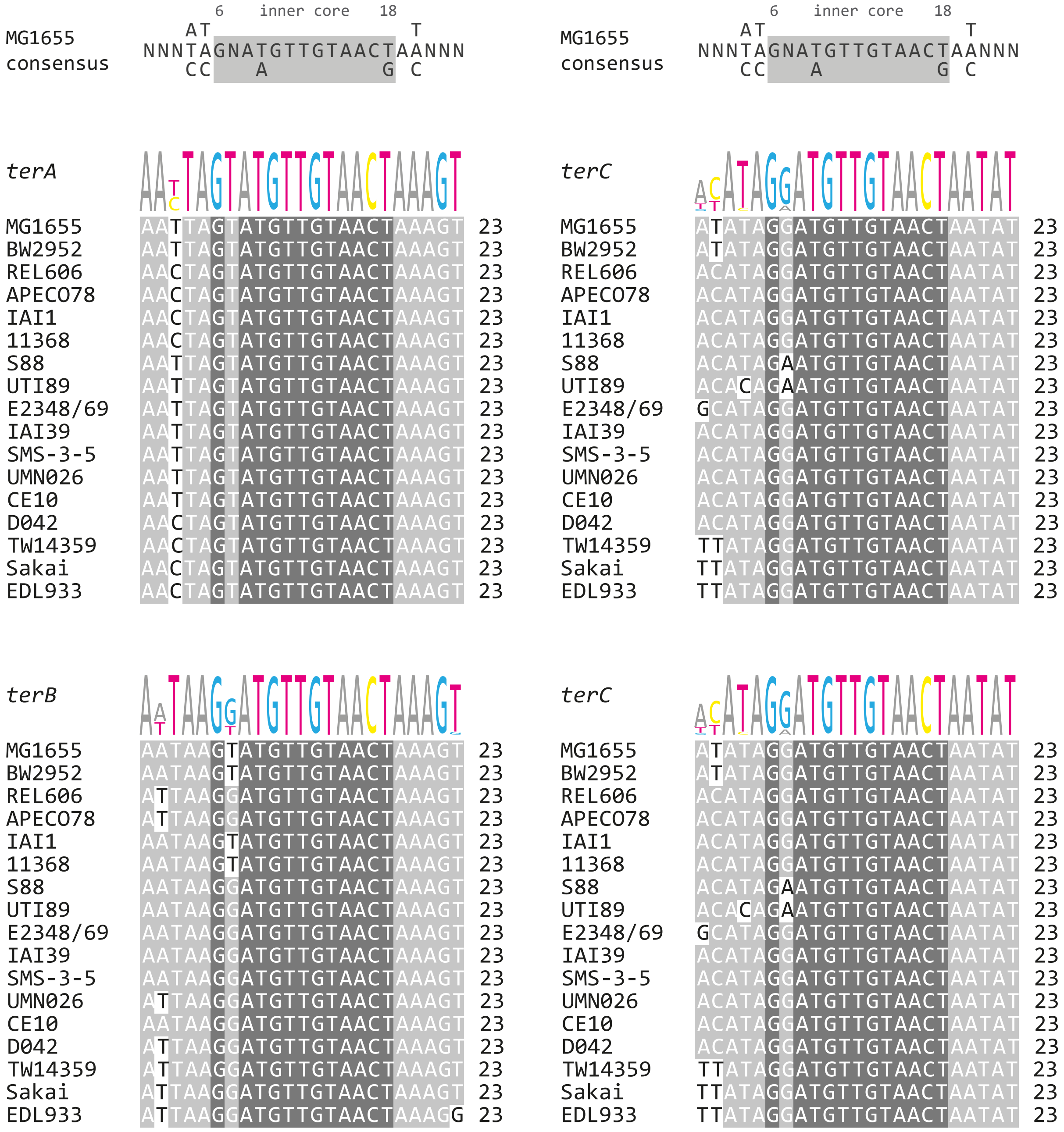

Figure S3. Sequence comparison of all *ter* sites across all phylogenetic groups from all *E. coli* genomes analysed, with the MG1655 sequences as reference. The consensus sequence of all *ter* sites in MG1655 is shown at the top. Base pairs 1 – 5, 7 and 19 – 23 show a high degree of variability and are shaded in a lighter grey. The highly conserved positions 6 and 8 – 18 are highlighted by a darker grey. Mismatches unlikely to interfere significantly with binding have a white background shading. Mismatches within the core conserved sequence that are likely to interfere with binding are highlighted in red. Relative nucleotide frequencies are shown above as scaled letters.

```
                      6   inner core   18
                 AT                     T
MG1655        NNNTAGNATGTTGTAACTAANNN
consensus        CC    A             G C
```

terE

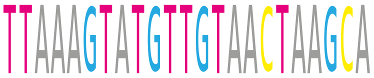

```
MG1655      TTAAAGTATGTTGTAACTAAGCA  23
BW2952      TTAAAGTATGTTGTAACTAAGCA  23
REL606      TTAAAGTATGTTGTAACTAAGCA  23
APECO78     TTAAAGTATGTTGTAACTAAGCA  23
IAI1        TTAAAGTATGTTGTAACTAAGCA  23
11368       TTAAAGTATGTTGTAACTAAGCA  23
S88         TTAAAGTATGTTGTAACTAAGCA  23
UTI89       TTAAAGTATGTTGTAACTAAGCA  23
E2348/69    TTAAAGTATGTTGTAACTAAGCA  23
IAI39       TTAAAGTATGTTGTAACTAAGCA  23
SMS-3-5     TTAAAGTATGTTGTAACTAAGCA  23
UMN026      TTAAAGTATGTTGTAACTAAGCA  23
CE10        TTAAAGTATGTTGTAACTAAGCA  23
D042        TTAAAGTATGTTGTAACTAAGCA  23
TW14359     TTAAAGTATGTTGTAACTAAGCA  23
Sakai       TTAAAGTATGTTGTAACTAAGCA  23
EDL933      TTAAAGTATGTTGTAACTAAGCA  23
```

terF

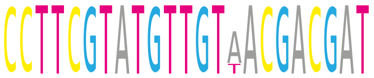

```
MG1655      CCTTCGTATGTTGTAACGACGAT  23
BW2952      CCTTCGTATGTTGTAACGACGAT  23
REL606      CCTTCGTATGTTGTAACGACGAT  23
APECO78     CCTTCGTATGTTGTAACGACGAT  23
IAI1        CCTTCGTATGTTGTAACGACGAT  23
11368       CCTTCGTATGTTGTAACGACGAT  23
S88         CCTTCGTATGTTGTAACGACGAT  23
UTI89       CCTTCGTATGTTGTAACGACGAT  23
E2348/69    CCTTCGTATGTTGTAACGACGAT  23
IAI39       CCTTCGTATGTTGTAACGACGAT  23
SMS-3-5     CCTTCGTATGTTGTAACGACGAT  23
UMN026      CCTTCGTATGTTGTTACGACGAT  23
CE10        CCTTCGTATGTTGTAACGACGAT  23
D042        CCTTCGTATGTTGTAACGACGAT  23
TW14359     CCTTCGTATGTTGTTACGACGAT  23
Sakai       CCTTCGTATGTTGTTACGACGAT  23
EDL933      CCTTCGTATGTTGTTACGACGAT  23
```


```
                      6   inner core   18
                 AT                     T
MG1655        NNNTAGNATGTTGTAACTAANNN
consensus        CC    A             G C
```


terG

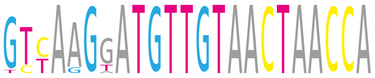

```
MG1655      GTCAAGGATGTTGTAACTAACCA  23
BW2952      GTCAAGGATGTTGTAACTAACCA  23
REL606      GTCAAGAATGTTGTAACTAACCA  23
APECO78     GTCAAGGATGTTGTAACTAACCA  23
IAI1        TTCAAGGATGTTGTAACTAACCA  23
11368       GTCAAGGATGTTGTAACTAACCA  23
S88         GTTAAGTATGTTGTAACTAACCA  23
UTI89       GTTAAGTATGTTGTAACTAACCA  23
E2348/69    GTTAAGTATGTTGTAACTAACCA  23
IAI39       GTCAGGGATGTTGTAACTAACCA  23
SMS-3-5     GTCAAGGATGTTGTAACTAACCA  23
UMN026      GTTAAGGATGTTGTAACTAACCA  23
CE10        GTCAGGGATGTTGTAACTAACCA  23
D042        GTCAAGGATGTTGTAACTAACCA  23
TW14359     GCTAAGGATGTTGTAACTAACCA  23
Sakai       GCTAAGGATGTTGTAACTAACCA  23
EDL933      GCTAAGGATGTTGTAACTAACCA  23
```

terH

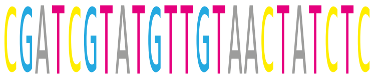

```
MG1655      CGATCGTATGTTGTAACTATCTC  23
BW2952      CGATCGTATGTTGTAACTATCTC  23
REL606      CGATCGTATGTTGTAACTATCTC  23
APECO78     CGATCGTATGTTGTAACTATCTC  23
IAI1        CGATCGTATGTTGTAACTATCTC  23
11368       CGATCGTATGTTGTAACTATCTC  23
S88         CGATCGTATGTTGTAACTATCTC  23
UTI89       CGATCGTATGTTGTAACTATCTC  23
E2348/69    CGATCGTATGTTGTAACTATCTC  23
IAI39       CGATCGTATGTTGTAACTATCTC  23
SMS-3-5     CGATCGTATGTTGTAACTATCTC  23
UMN026      CGATCGTATGTTGTAACTATCTC  23
CE10        CGATCGTATGTTGTAACTATCTC  23
D042        CGATCGTATGTTGTAACTATCTC  23
TW14359     CGATCGTATGTTGTAACTATCTC  23
Sakai       CGATCGTATGTTGTAACTATCTC  23
EDL933      CGATCGTATGTTGTAACTATCTC  23
```

Figure S3 continued.

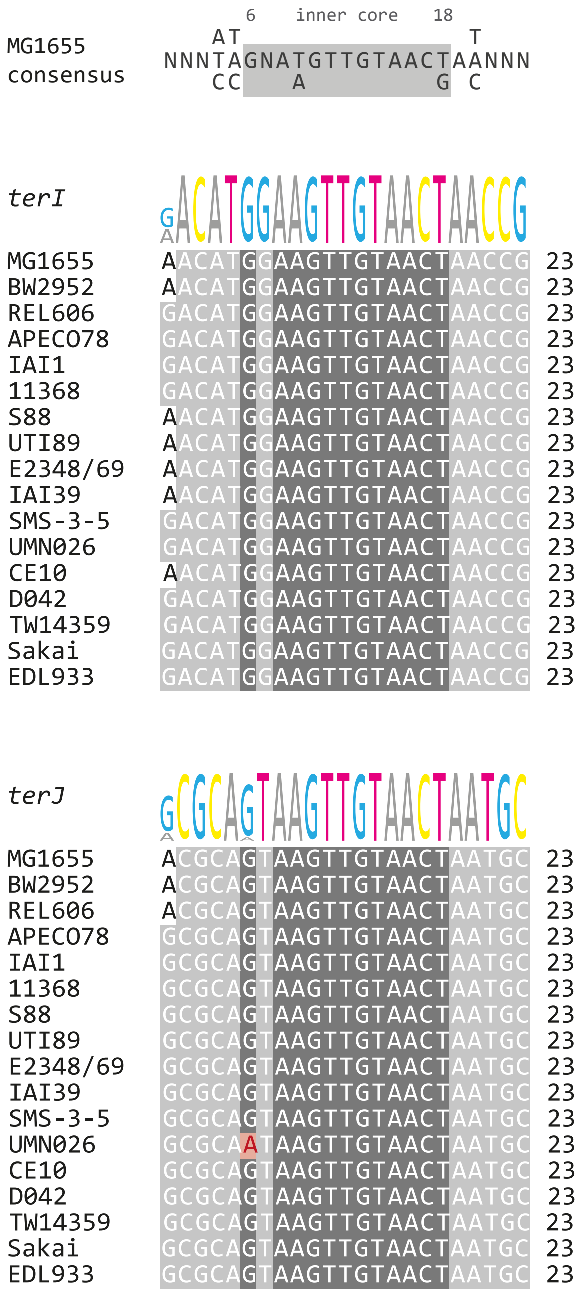

Figure S3 continued.

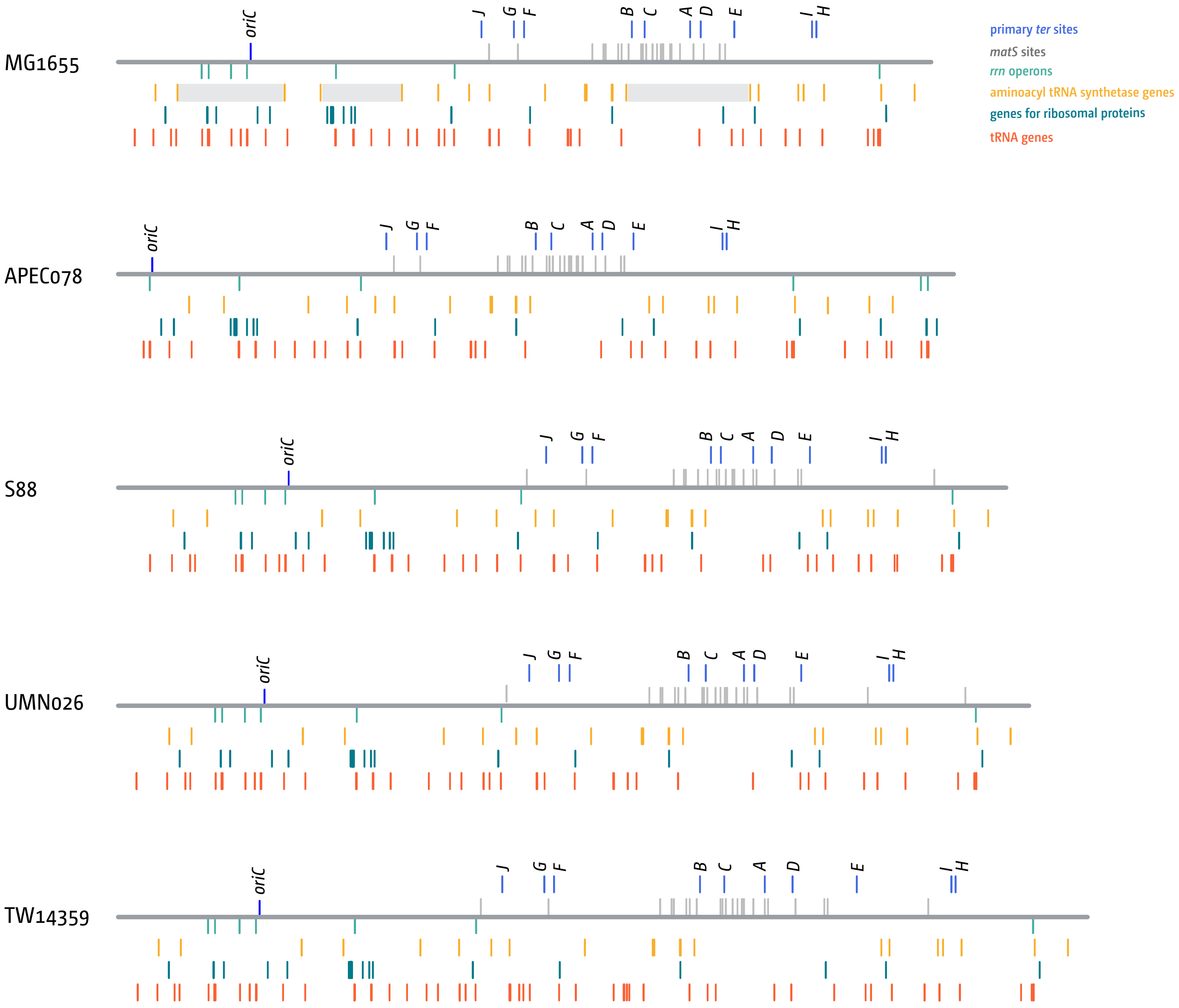

Figure S4. Location and distribution of genes and genetic markers involved in termination of DNA replication as well as translation. Above the line representing the chromosome the origin is shown, together with all primary *ter* sites (blue) and all *matS* sites (grey), which, if bound by MatP, define the chromosomal Ter domain. Below are shown the *rrn* operons (green), genes coding for aminocyl tRNA synthetases (yellow), genes encoding ribosomal proteins (cyan) and tRNA genes (orange). Significant gaps in the distributions of aminocyl tRNA synthetase genes are shown via a grey background for MG1655 (see main text for details).

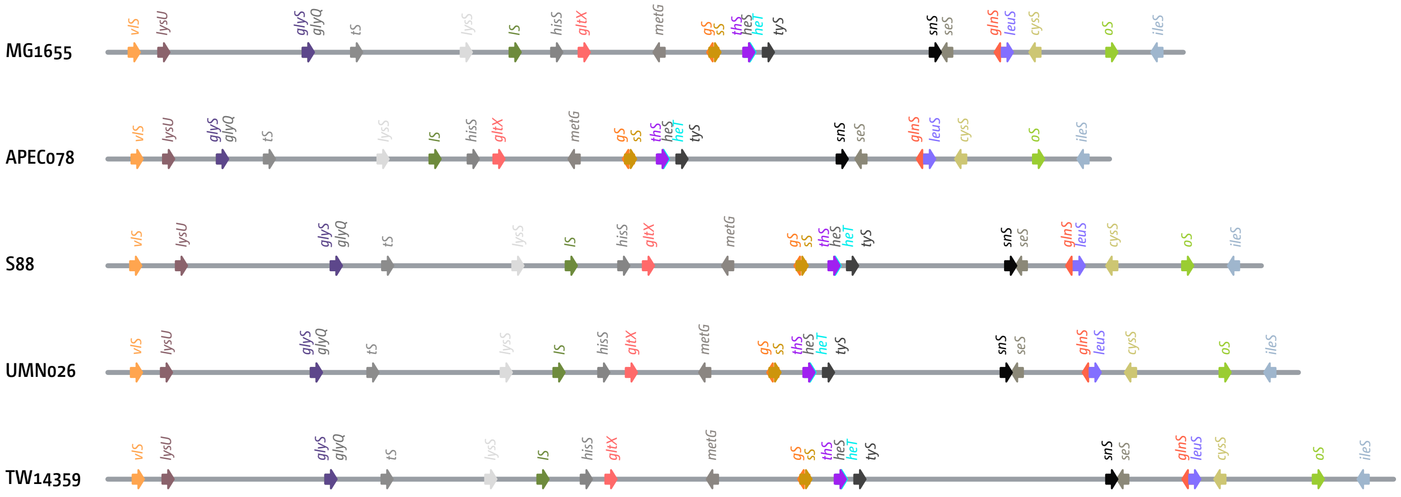

Figure S5. Location and relative order of aminoacyl tRNA synthetase genes within the chromosomes of the *E. coli* phylogroups A (MG1655), B1 (APEC078), B2 (S88), D (UMN026) and E (TW14359). Every gene within a certain genome has a specific colour, which highlights similarities in position across all genomes. Gene names are given above. Transcriptional directionality of the genes is highlighted by an arrow.

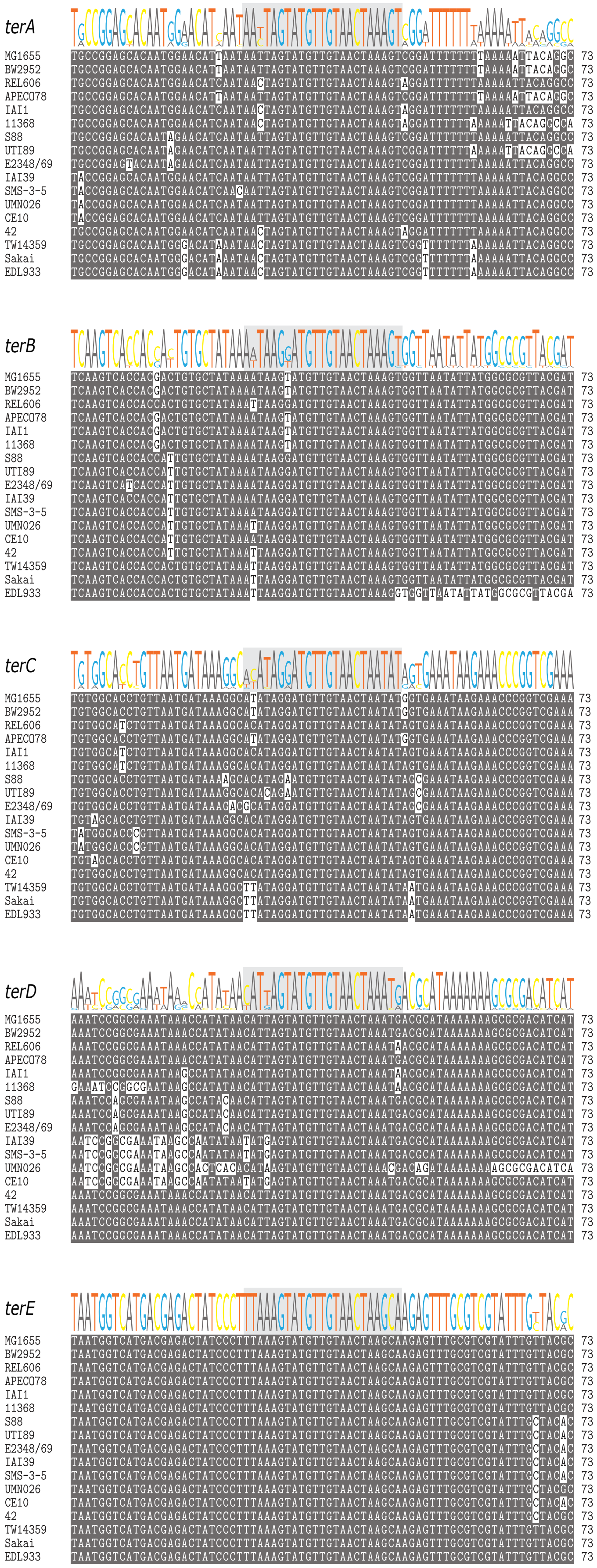

Figure S6. Sequence comparison of all *ter* sites, including 25 bp upstream as well as downstream, across all phylogenetic groups from all *E. coli* genomes analysed, with the MG1655 sequences as reference. The *ter* sequence is highlighted by a light grey box in the nucleotide frequencies at the top of each comparison. All conserved positions are highlighted by a darker grey, while mismatches are shown as a black letter on a white background. Relative nucleotide frequencies are shown above as scaled letters.

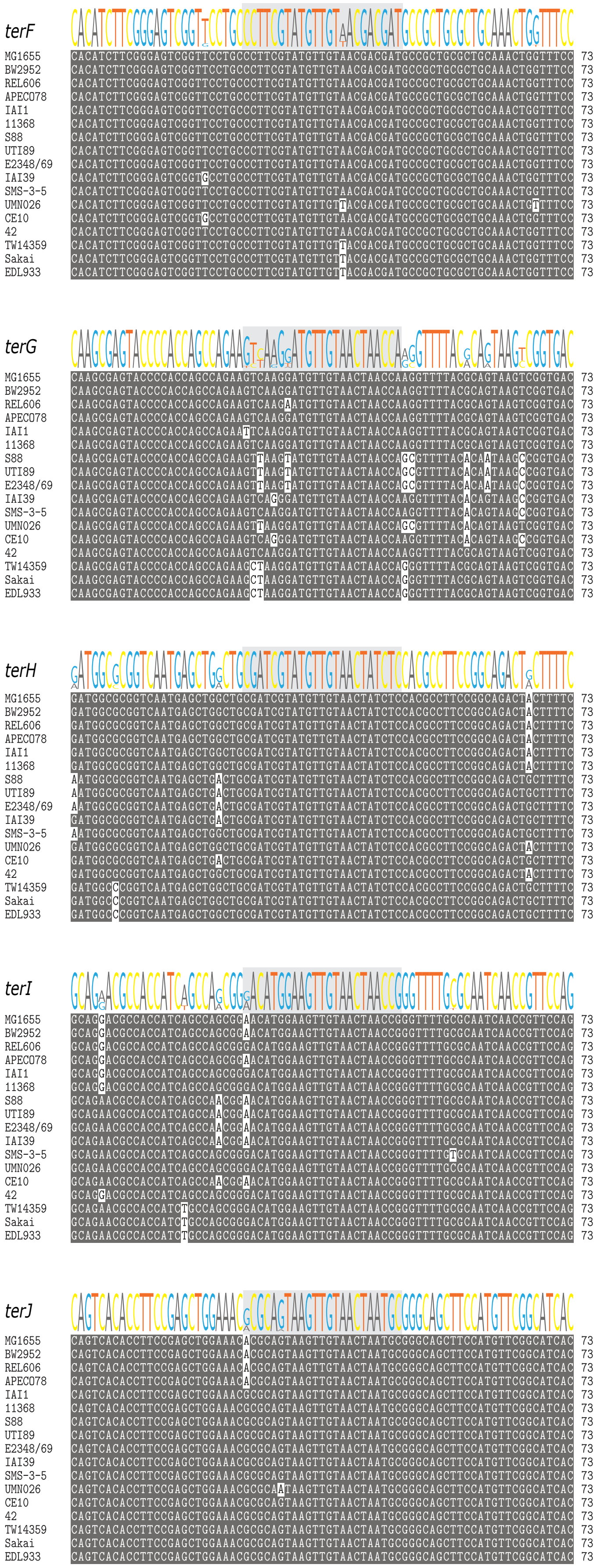

Figure S6 continued.

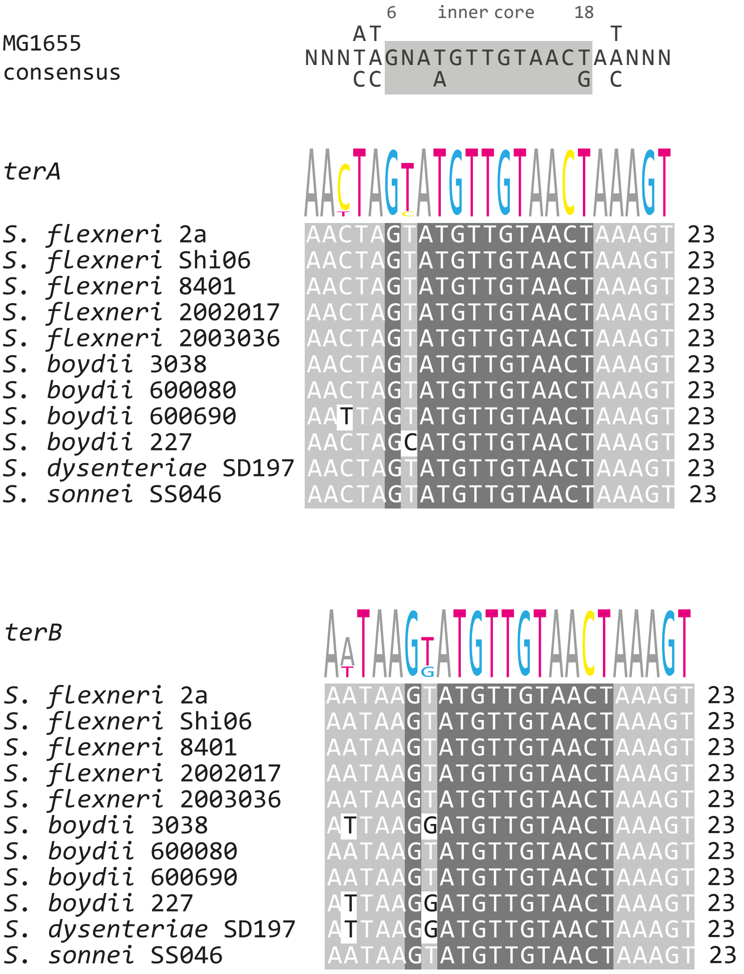

Figure S7. Sequence comparison of all *ter* sites across all serotypes from all *Shigella* genomes analysed. The *ter* consensus sequence in MG1655 is shown at the top. Base pairs 1 – 5, 7 and 19 – 23 show a high degree of variability and are shaded in a lighter grey. The highly conserved positions 6 and 8 – 18 are highlighted by a darker grey. Mismatches unlikely to interfere significantly with binding have a white background shading. Mismatches within the core conserved sequence that are likely to interfere with binding are highlighted in red. Relative nucleotide frequencies are shown above as scaled letters. For *S. boydii* 227 we were unable to find a sequence that resembles *terC* (Figure 6) and for this reason the 227 genome is missing in the *terC* analysis.

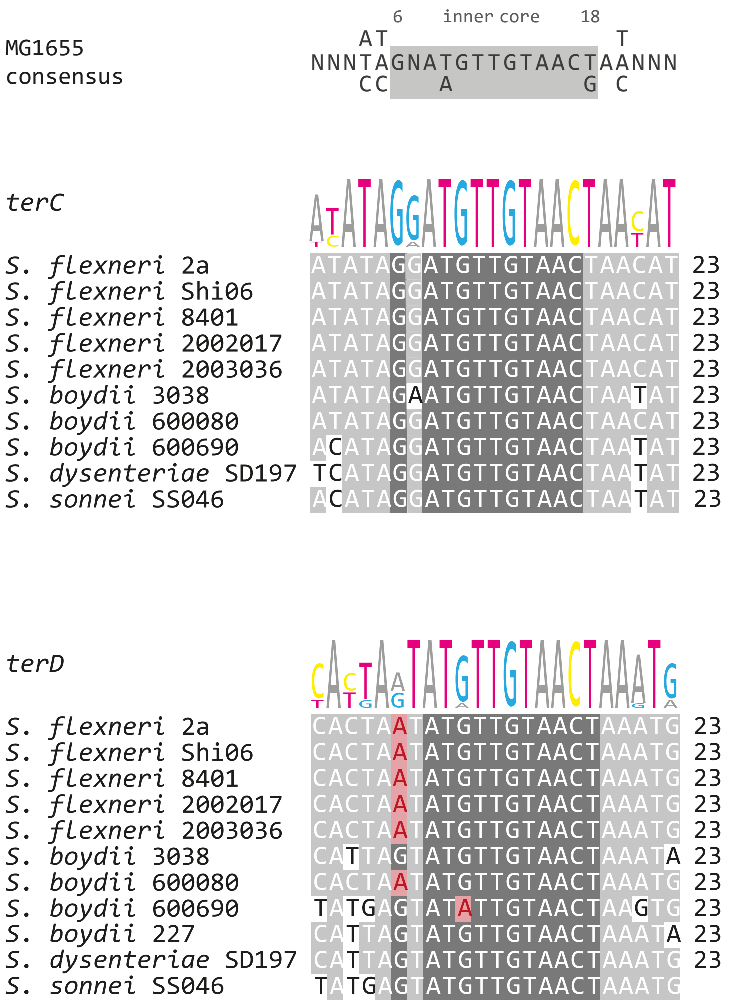

Figure S7 continued.

SUPPLEMENTARY TABLES

Table S1. *Escherichia coli* K12 strains

Strain number	Relevant Genotype[a]	Source
**MG1655 derivatives**		
MG1655	F^- *rph-1*	[1]
AM1775	Δ*tus::cat*	[2]
JD1221	Δ*dif::cat dif*^{ectopic}-<*kan*>	JD1217 × P1.JD1219 to Km^r
JD1217	Δ*dif::cat*	MG1655 × P1.JD1212 to Cm^r
JD1212	Δ*dif::cat*[b]	This study
JD1219	Δ*dif::cat dif*^{ectopic}-<*kan*>[b]	This study

a – Only the relevant additional genotype of the derivatives is shown. The abbreviations kan and cat refer to insertions conferring resistance to kanamycin (Km^r) and chloramphenicol (Cm^r), respectively. '<>' indicates the use of *frt* sites, where *frt* stands for the 34 bp recognition site of the FLP/*frt* site-directed recombination system. Thus, <*kan*> refers to a kanamycin marker flanked by an *frt* site either side.
b – Integration was achieved via the PCR-based one-step chromosomal integration procedure described by Datsenko and Wanner [3]. For *dif*^{ectopic}-<*kan*> the *dif* site, linked to a kanamycin resistance cassette, was integrated between *ydgA* and *gusC*, just outside of *terB*. Primer sequences of integration constructs are available upon request.

Table S2. List of all *E. coli* genomes used in this study, including NCBI nucleotide database access links.

Organism	Strain	Phylogroup	NCBI accession number
E. coli	MG1655	A	NC_000913.3
	BW2952	A	CP001396
	REL606	A	CP000819.1
	APEC078	B1	NC_020163.1
	IAI1	B1	NC_011741.1
	11368	B1	NC_013361.1
	S88	B2	NC_011742.1
	UTI89	B2	NC_007946.1
	E2348	B2	NC_011601.1
	UMN026	D	NC_011751.1
	IAI39	D	NC_011750.1
	SMS-3-5	D	NC_010498.1
	CE10	D	NC_017646.1
	042	D	NZ_CP042934.2
	TW14359	E	NC_013008.1
	Sakai	E	NC_002695.2
	EDL933	E	NC_002655.2

Table S3. List of all *Shigella, Salmonella* and *Klebsiella* genomes used in this study, including NCBI nucleotide database access links.

Organism	Strain	Link
Shigella	*flexneri* 2a str. 301	AE005674.2
	flexneri Shi06HN006	CP004057.1
	flexneri 5 str. 8401	CP000266.1
	flexneri 2002017	CP001383.1
	flexneri 2003036	CP004056.1
	boydii CDC 3083-94	CP001063.1
	boydii 600080	CP049606.1
	boydii 600690	CP049278.1
	boydii Sb227	CP000036.1
	dysenteriae Sd197	NC_007606.1
	sonnei Ss046	CP000038.1
Salmonella	*enterica* subsp. *enterica*, serovar Typhimurium DT104	HF937208.1
	enterica strain FDAARGOS_878	NZ_CP065718
Klebsiella	*variicola* strain DX120E	CP009274
	pneumoniae subsp. *pneumoniae* HS11286	CP003200

Table S4. Location of the 10 major *ter* sites in relation to predicted open reading frames within the 5 main *E. coli* phylogenetic groups

ter site	MG1655 (A)	APEC078 (B1)	S88 (B2)	UMN026 (D)	TW14359 (E)
terA[a]	outside ORF	outside ORF	*partial overlap*	*partial overlap*	*partial overlap*
terB	outside ORF	outside ORF	outside ORF	outside ORF	outside ORF
terC	outside ORF	outside ORF	outside ORF	outside ORF	outside ORF
terD	outside ORF	outside ORF	outside ORF	outside ORF	outside ORF
terE	outside ORF	outside ORF	outside ORF	outside ORF	outside ORF
terF	inside ORF	inside ORF	inside ORF	inside ORF	inside ORF
terG	inside ORF	inside ORF	inside ORF	inside ORF	inside ORF
terH	inside ORF	inside ORF	inside ORF	inside ORF	inside ORF
terI	inside ORF	inside ORF	inside ORF	inside ORF	inside ORF
terJ	inside ORF	inside ORF	inside ORF	inside ORF	inside ORF

a – the innermost *ter* sites are highlighted in red, while outer *ter* sites are in dark grey.

Table S5. Doubling times of *E. coli* strains with the chromosome dimer resolution site *dif* in its native or an ectopic location.

Strain Background	Doubling Time [min]	SD
MG1655	21.8	± 1.3
dif^{ectopic}	26.7[a]	± 3.9

a – the doubling time of the *dif*^{ectopic} derivative is significantly different to the doubling time of wild type cells according to a Wilcoxon signed-rank test (p = 0.05).

SUPPLEMENTARY REFERENCES

[1] Bachmann, B J. Derivations and Genotypes of Some Mutant Derivatives of *Escherichia coli* K-12. Escherichia Coli Salmonella Cell. Mol. Biol. Second Edition, ASM Press; 1996.

[2] Rudolph CJ, Upton AL, Stockum A, Nieduszynski CA, Lloyd RG. Avoiding chromosome pathology when replication forks collide. Nature 2013;500:608–11. https://doi.org/10.1038/nature12312.

[3] Datsenko KA, Wanner BL. One-step inactivation of chromosomal genes in *Escherichia coli* K-12 using PCR products. Proc Natl Acad Sci U S A 2000;97:6640–5. https://doi.org/10.1073/pnas.120163297.

[4] Castillo F, Benmohamed A, Szatmari G. Xer Site Specific Recombination: Double and Single Recombinase Systems. Front Microbiol 2017;8:453. https://doi.org/10.3389/fmicb.2017.00453.
